# Development of an oral reference dose for the perfluorinated compound GenX

**DOI:** 10.1002/jat.3812

**Published:** 2019-06-18

**Authors:** Chad M. Thompson, Seneca E. Fitch, Caroline Ring, William Rish, John M. Cullen, Laurie C. Haws

**Affiliations:** ^1^ ToxStrategies, Inc. Katy Texas; ^2^ ToxStrategies, Inc. Austin Texas; ^3^ ToxStrategies, Inc. Ashville North Carolina; ^4^ College of Veterinary Medicine North Carolina State University Raleigh North Carolina

**Keywords:** benchmark dose (BMD) modeling, GenX, per‐ and polyfluoroalkyl substances (PFAS), reference dose (RfD), risk assessment

## Abstract

Ammonium 2,3,3,3‐tetrafluoro‐2‐(heptafluoropropoxy)‐propanoate, also known as GenX, is a processing aid used in the manufacture of fluoropolymers. GenX is one of several chemistries developed as an alternative to long‐chain poly‐fluoroalkyl substances, which tend to have long clearance half‐lives and are environmentally persistent. Unlike poly‐fluoroalkyl substances, GenX has more rapid clearance, but has been detected in US and international water sources. There are currently no federal drinking water standards for GenX in the USA; therefore, we developed a non‐cancer oral reference dose (RfD) for GenX based on available repeated dose studies. The review of the available data indicate that GenX is unlikely to be genotoxic. A combination of traditional frequentist benchmark dose models and Bayesian benchmark dose models were used derive relevant points of departure from mammalian toxicity studies. In addition, deterministic and probabilistic RfD values were developed using available tools and regulatory guidance. The two approaches resulted in a narrow range of RfD values for liver lesions observed in a 2‐year bioassay in rats (0.01–0.02 mg/kg/day). The probabilistic approach resulted in the lower, i.e., more conservative RfD. The probabilistic RfD of 0.01 mg/kg/day results in a maximum contaminant level goal of 70 ppb. It is anticipated that these values, along with the hazard identification and dose‐response modeling described herein, should be informative for risk assessors and regulators interested in setting health‐protective drinking water guideline values for GenX.

## Abstract 2

Ammonium 2,3,3,3‐tetrafluoro‐2‐(heptafluoropropoxy)‐propanoate, also known as GenX, is a processing aid used in the manufacture of fluoropolymers. There are currently no federal drinking water standards for GenX in the USA. Frequentist benchmark dose models and Bayesian benchmark dose models were used to derive points of departure from mammalian toxicity studies. Deterministic and probabilistic reference dose values were developed and resulted in a narrow range of values (0.01‐0.02 mg/kg/day). The lower reference dose results in a maximum contaminant level goal of 70 ppb.

## 1 INTRODUCTION

GenX is the trade name for ammonium 2,3,3,3‐tetrafluoro‐2‐(heptafluoropropoxy)‐propanoate (CAS no. 62037‐80‐3; molecular weight 347.08), which is a short‐chain perfluoroether carboxylic acid compound that has some structural similarity to perfluoroalkyl carboxylic acids (PFCAs) and is a subclass of perfluoroalkyl acids. Collectively, these compounds are broadly referred to as per‐ and polyfluoroalkyl substances (PFAS). PFAS are anthropogenic organic compounds that have been used for decades in a wide variety of consumer and industrial products including: oil‐resistant coatings for food packaging; water‐ and stain‐resistant coatings for clothing, carpets and upholstery; fire‐fighting foams; non‐stick coatings on cookware (e.g., Teflon); personal care products (e.g., dental floss, cosmetics, sunscreen); and industrial surfactants and emulsifiers (ATSDR, 2018; Buck et al., 2011; Lau et al., 2007). The physical properties that make PFAS useful in these commercial and industrial applications also make them resistant to biodegradation, photo‐oxidation, direct photolysis and hydrolysis—resulting in persistence in the environment (Lau et al., 2007). Because of the long‐term and widespread production and use of PFAS, these compounds have been detected in the environment, as well as in the tissues of wildlife and humans (ATSDR, 2018; Buck et al., 2011; Lau et al., 2007).

The two most widely‐known, well‐studied, and most often reported and discussed PFAS are perfluorooctanoic acid (PFOA) and perfluorooctane sulfate (PFOS), both of which are considered “long‐chain” PFAS (Buck et al., 2011). The term “long‐chain” refers to PFAS with eight or more carbons, of which at least seven or more are perfluorinated carbons, and perfluoroalkane sulfonates with six or more carbons, of which at least six or more are perfluorinated carbons (Buck et al., 2011). Because of concerns about the environmental persistence and toxicity of the long‐chain PFAS and perfluoroalkane sulfonates, and in particular, PFOA and PFOS, there have been numerous global regulatory initiatives aimed at eliminating the production and use of these compounds. In 2002, the major manufacturer (3M) phased out production of PFOS; in 2006, the US EPA initiated the PFOA Stewardship Program, which was aimed at eliminating emissions and product content of PFOA by 2015; in 2006, the European Union issued a directive restricting the use of perfluorooctane sulfonates; in 2009, the Stockholm Convention included PFOS on the list of persistent organic pollutants and identified it as an Annex B substance; in 2010, the Canadian environmental and health agencies reached an agreement with manufacturers to restrict long‐chain PFAS in products (Buck et al., 2011; Lau et al., 2007). However, because of the usefulness of these compounds across a broad array of consumer and industrial applications, efforts were undertaken to find replacements with more favorable environmental and biological properties (Gannon et al., 2016). Scientific evidence at that time suggested that short‐chain PFAS, such as GenX, were a less toxic, less bioaccumulative alternative to PFOA (Goecke‐Flora & Reo, 1996; Kudo et al., 2001). As a result, GenX replaced PFOA as a processing aid in the production of fluoropolymers (Gannon et al., 2016).

In recent years, there has been growing concern about potential human exposure to short‐chain PFAS, driven in part by the detection of GenX in the Cape Fear River, as well as in the finished drinking water from this river (Hopkins, Sun, DeWitt, & Knappe, 2018; Strynar et al., 2015; Sun et al., 2016). To date, the US EPA has yet to finalize toxicity values for GenX, nor has it established drinking water guidelines or standards. As such, the goal of this current work is to develop an oral reference dose (RfD) based on the best available science and consideration of the weight of evidence to provide critical information necessary to support risk‐based decisions aimed at addressing potential human exposures to GenX.

## 2 MATERIALS AND METHODS

**Figure 1:**
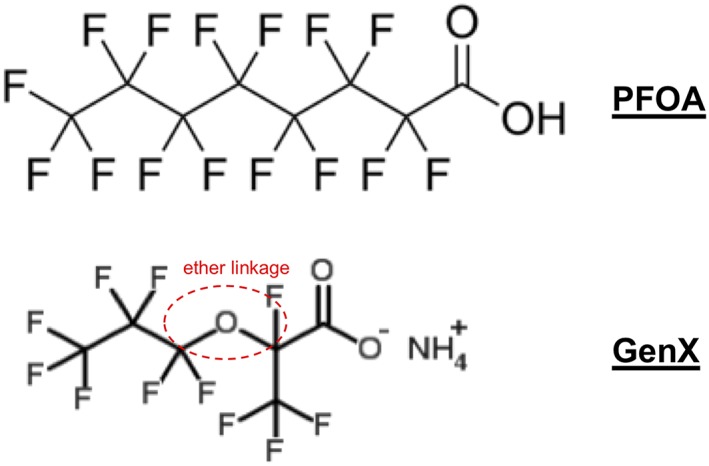
Comparison of 6‐carbon perfluoroether carboxylic acid GenX to the 8‐carbon perfluoroalkyl carboxylic acid, PFOA [Colour figure can be viewed at wileyonlinelibrary.com]

As already noted, GenX is the trade name for ammonium 2,3,3,3‐tetrafluoro‐2‐(heptafluoropropoxy)‐propanoate (CAS no. 62037‐80‐3). Unlike perfluoroalkyl acids, perfluoroether carboxylic acids such as GenX contain an ether linkage within their carbon chain (Figure 1). Other names for GenX include HFPO‐DA (hexafluoropropylene oxide‐dimer acid). In water, the ammonium salt readily dissociates leaving the ion 2,3,3,3‐tetrafluoro‐2‐(heptafluoropropoxy)‐propanoic acid (CAS no. 13252‐13‐6). This anion results from the dissociation of the neutral acid. Herein, both forms will be referred to as GenX.

### 2.1 Data selection

Data sources used for deriving toxicity criteria for GenX are all publicly available, although some of the data sources are not indexed in popular databases such as PubMed. (All non‐published studies conducted by DuPont/Chemours are available in the US EPA HERO database at: https://hero.epa.gov/hero/index.cfm/project/page/page/1/rows/10/sort/year%20desc/format/list/project_id/2627/criteria_all/DuPont/usage_searchType/any.) In addition to reviewing original study reports conducted by contract research laboratories for Chemours™, a literature search was conducted to identify any additional studies. The following search terms were used to search PubMed and Embase for data relevant for the derivation of toxicity criteria protective of human health: GenX, HFPO‐DA 2,3,3,3‐tetrafluoro‐2‐(heptafluoropropoxy)‐propanoate, 2,3,3,3‐tetrafluoro‐2‐(heptafluoropro‐poxy) propionic acid, propanoic acid, 2,3,3,3‐tetrafluoro‐2‐(1,1,2,2,3,3,3‐heptafluoropropoxy)‐, ammonium salt (1:1), heptafluoropropyl 1,2,2,2,‐tetrafluoroethyl ether, propane, 1,1,1,2,2,3,3‐heptafluoro‐3‐(1,2,2,2‐tetrafluoroethoxy) and corresponding CAS nos 62037‐80‐3, 13252‐13‐6 and 3330‐15‐2. Search syntax was not limited by health effect or outcome and all literature was reviewed. Additional searches using the above key words were also performed via Google Scholar and ToxPlanet to capture gray literature and publications from government agencies. Initial searches were performed on May 7, 2018 and updated on October 11, 2018 to ensure all potentially relevant articles were captured.

### 2.2 Dose‐response analysis

Dose‐response modeling was conducted with US EPA's Benchmark Dose Software (BMDS) v.2.7 and 3.0, using the suite of dichotomous and continuous models. BMDS v3.0 was released after initial analyses were run in BMDS 2.7. Bayesian models in BMDS v3.0 were used as input for probabilistic RfD (pRfD) derivation. Benchmark response (BMR) values were 10% extra risk for dichotomous datasets, 5% extra risk for some developmental endpoints, and 1 SD for continuous datasets per typical US EPA practice (US EPA, 2012). These models were used to obtain benchmark dose (BMD) values and their corresponding 95% lower confidence limit (BMDL) values. Model fits were judged using criteria such as *P*‐values, scaled residuals, Akaike information criterion, parsimony and visual inspection (US EPA, 2012). For the final RfD derivation (see below), the Bayesian model averages for the BMD_10_ and BMDL_10_ were obtained using BMDS 3.0. Model plots shown herein are from v2.7 because plots from v3.0 do not currently include error bars.

### 2.3 Reference dose derivation

RfD values for GenX were derived using deterministic and probabilistic approaches. For the deterministic RfD values, the BMDL values from animal toxicity studies were converted to human equivalent dose (HED, mg/kg/day) values using allometric scaling per standard US EPA practice (US EPA, 2002; US EPA, 2011). The HED values were then adjusted by a combination of uncertainty factors (UFs) per US EPA guidance (US EPA, 2002), including the interspecies UF (UF_A_; unitless) and intraspecies UF (UF_H_; unitless). Additional UFs considered included use of data from less‐than‐lifetime (i.e. subchronic) studies (UF_S_; unitless) and overall completeness of database (UF_D_; unitless). The deterministic RfD calculation is as follows

$$\mathrm{RfD}\;\left(\mathrm{mg}/\mathrm{kg}/\mathrm{day}\right)=\mathrm{HED}÷\left[{\mathrm{UF}}_{A}\times {\mathrm{UF}}_{H}\times {\mathrm{UF}}_{S}\times {\mathrm{UF}}_{D}\right]$$

A probabilistic RfD (pRfD) was also developed using recently described methods (Chiu & Slob, 2015; Chiu et al., 2018). To facilitate the derivation of a pRfD, the R code developed to perform the analyses in Chiu et al. (2018) was obtained (https://github.com/wachiuphd/Probabilistic‐RfD), reviewed and adapted. In probabilistic terminology, the critical effect size (or magnitude) is equivalent to the BMR and is denoted as magnitude (M) (Chiu & Slob, 2015). Any BMD from an animal study has an associated uncertainty distribution that can be obtained from the confidence limits on the BMD; the animal dose can thus be represented by a random variable (AD_M_) that obeys this BMD uncertainty distribution. For example, an AD_10_ would represent a BMD_10_ obtained from modeling animal data. To convert the probabilistic animal dose to a corresponding probabilistic human dose, two probabilistic adjustment factors are applied to the AD_M_, representing allometric scaling (AF_BW_) and remaining interspecies toxicokinetic and toxicodynamic adjustment factor (AF_TK/TD_). AF_BW_ and AF_TK/TD_ are random variables representing the possible conversion factors between an animal dose and the corresponding human dose. They obey default log‐normal distributions developed from historical data (Chiu et al., 2018). Applying these probabilistic adjustment factors to the probabilistic AD_M_ results in another random variable representing the equivalent dose for the median human, denoted as the median human distribution (HD_M_^50%^). For an AD_M_ based on a BMR of 10% extra risk (i.e., AD_10_), the associated HD_10_^50%^ represents the dose at which 50% of the human population has a 10% extra risk of developing the effect of interest. To protect potentially sensitive individuals, a human variability adjustment factor (AF_H_^I^) is applied to the HD_M_^50%^. AF_H_^I^ represents the ratio of the dose for the median human and the equipotent dose for the most‐sensitive *I*% of humans (HD_M_^I^), where *I* is the target population incidence for the non‐cancer effect. AF_H_^I^ is itself uncertain, and obeys a default log‐normal distribution based on historical data (Chiu et al., 2018). We follow the approach of Chiu and colleagues in selecting *I* = 1% as a reasonably conservative target incidence (Chiu et al., 2018; Chiu & Slob, 2015). Applying AF_H_^I=1%^ to the HD_10_^50%^ results in a random variable representing the dose at which the most‐sensitive 1% of the human population has a 10% extra risk of developing the effect of interest: the HD_10_^1%^. The fifth percentile of the distribution of the HD_10_^1%^ is designated the pRfD per Chiu et al. (2018).

Notably, Chiu et al. (2018) did not apply an adjustment factor corresponding to the UF_D_ in their derivation of pRfDs, in part, because no database has been developed from which to infer a distribution for such an adjustment factor. Without applying any database UF, the pRfD relates only to the specific effect for which it was evaluated, rather than relating to the general probability of any deleterious effect (Chiu et al., 2018). However, to remain consistent with current risk assessment practice, we applied a non‐probabilistic UF_D_ to the fifth percentile of the HD_10_^1%^.

The pRfD calculation herein is as follows:

$$\begin{array}{l} {{\mathrm{HD}}_{10}}^{50\%}={\mathrm{AD}}_{10}÷\left({\mathrm{AF}}_{\mathrm{BW}}x\;{\mathrm{AF}}_{\mathrm{TD}}\right)\\ {{\mathrm{HD}}_{10}}^{1\%}={{\mathrm{HD}}_{10}}^{50\%}÷{\mathrm{AF}}_{H}\\ \text{pRfD}=\left(5\mathrm{th}\;\text{percentile of}\;{{\mathrm{HD}}_{10}}^{1\%}\right)÷{\mathrm{UF}}_{D} \end{array}$$

where AD_10_ is a random variable representing the animal BMD; HD_10_^50%^ a random variable representing the dose at which 50% of the human population has a 10% extra risk of developing the effect of interest; HD_10_^1%^ a random variable representing the equipotent dose to the most‐sensitive 1% of humans; AF_BW_ a random variable representing the allometric scaling factor; AF_TK/TD_ a random variable representing the adjustment factor for remaining differences in toxicokinetics and toxicodynamics between animals and humans after applying allometric scaling; AF_H_ a random variable representing the adjustment factor for human variability; UF_D_ a non‐random variable representing uncertainty in the completeness of the database (unitless). With the exception of the UFD, all adjustment factors obeyed the default, independent log‐normal distributions defined in Chiu et al. (2018).

### 2.4 Maximum contaminant level goal

The percentage of exposure to GenX accounted for by drinking water exposure, i.e., the relative source contribution (RSC), was set to the US EPA default value of 20% (US EPA, 2000). As outlined in 40 CFR, Parts 141 and 142 (US EPA, 1992), the following equation and input parameters are applied in deriving a maximum contaminant level goal (MCLG):

$$\text{MCLG}\;=\left(\mathrm{RfD},\;,\times ,\;,\text{BW},\;,\times ,\;,\text{RSC}\right)\;/\;\text{drinking water intake}$$

where bodyweight is at 70 kg (adults), drinking water intake at 2 L/day and default relative source contribution at 0.2% or 20%

### 2.5 Re‐evaluation of hepatocellular single cell necrosis in male mice

Multiple studies of GenX have diagnosed hepatocyte “single cell necrosis” in the mouse liver (Edwards, 2010b; MacKenzie, 2010). Recent guidance indicates that hepatocellular death previously diagnosed broadly as single cell necrosis should be histologically distinguished as apoptosis or necrosis (Elmore et al., 2016). Because such diagnoses might have implications for mode of action (MOA), hematoxylin and eosin (H&E)‐stained slides of liver sections from male mice exposed to GenX (Edwards, 2010b) were re‐evaluated by a board‐certified veterinary pathologist (J.M.C.). Emphasis was placed on evaluating the samples for the presence and type of individual hepatocyte necrosis. The two terms recommended for hepatocyte death were apoptosis and necrosis based on the proposed nomenclature from the Terminology Recommendations from the INHAND Apoptosis/Necrosis Working Group (Tables S1 and S2; see Supporting Information). The STP INHAND Nomenclature for Non‐neoplastic Findings of the Rodent Liver was also consulted for final diagnostic nomenclature (Thoolen et al., 2010). To assess hepatocyte single cell necrosis and mitosis, cells were tallied across 10 fields (20× objective). Severity was scored as follows: grade 0 = no evident change; grade 1 = minimal (present in 1‐10 hepatocytes/10, 20× fields); grade 2 = mild (present in 11‐40 hepatocytes/10, 20× fields); grade 3 = moderate (present in 41‐80 hepatocytes/10, 20× fields).

## 3 RESULTS

### 3.1 Literature search

In the initial literature search (May 07, 2018), PubMed and Embase queries retrieved 31 and 42 citations, respectively, to be reviewed for relevance to risk assessment. Records from both databases were combined, de‐duplicated and screened by title and abstract to identify publications for full‐text screening. Relevant data sets were identified and utilized in the analyses herein. A follow‐up search performed on October 11, 2018 returned an additional six studies in PubMed and five studies in Embase. Of these, none were considered relevant and were not included in the data evaluation; for example, some studies were about the “Generation X” demographic.

### 3.2 Hazard ID

#### 3.2.1 Toxicokinetics

Pharmacokinetic studies on GenX have been conducted in rats, mice and monkeys (Gannon et al., 2016). Female rats demonstrate clearances rates for GenX that are approximately 10‐fold higher than male rats. As will be discussed in subsequent sections, male rats are more sensitive to GenX than female rats, which is likely due to reduced clearance. Unlike rats, mice do not demonstrate large sex differences in GenX clearance. Comparing male rats and mice, the latter have slightly longer half‐lives for GenX (Gannon et al., 2016). The clearance rates in male and female monkeys were both similar to male rats. Broadly, Gannon et al. (2016) concluded that the pharmacokinetics in monkeys were more similar to rats than mice.

Human data recently released by the state of North Carolina indicate that GenX was found in “most” of 198 tap water samples taken from homes in New Hanover County, NC between October and December of 2017, with a median concentration of 50 ppt. (https://chhe.research.ncsu.edu/coec/projects/genx/the‐genx‐exposure‐study/). Blood and urine was collected from 310 participants in November of 2017 and 35 participants in May of 2018 (56 children were included). With a reporting limit of 2 ppb, GenX was not found in the blood of any subjects, including 30 people said to be living near a source plant. At the time that this publication was prepared, the urine data have not been released. Overall, the absence of GenX in blood samples of residents presumably exposed to GenX in tap water indicates that GenX does not have a long half‐life like some historically used PFAS.

#### 3.2.2 Subacute and subchronic toxicity studies

Subacute 28‐day toxicity studies, conducted in accordance with Organization for Economic Cooperation and Development (OECD) Test Guideline 407 and performed under Good Laboratory Practice (GLP) conditions, are available in both rats and mice (Haas, 2008a, b). (All non‐published studies conducted by DuPont/Chemours are available in the US EPA HERO database at: https://hero.epa.gov/hero/index.cfm/project/page/page/1/rows/10/sort/year%20desc/format/list/project_id/2627/criteria_all/DuPont/usage_searchType/any.) These 28‐day studies will not be discussed in detail, as there are longer‐term subchronic studies (i.e., 90‐day studies) available, which are more relevant for setting chronic toxicity values. However, both 28‐day studies included assessments of the ability of GenX to act as a peroxisome proliferator‐activated receptor‐α (PPARα) activator. In rats, males were exposed to 0.3, 3 and 30 mg/kg GenX by oral gavage and females were exposed to 3, 30 and 300 mg/kg. Hepatic β‐oxidation activity was increased in males at all doses, and in females at 30 and 300 mg/kg. Enzyme activity returned to control levels after 28 days of recovery (Haas, 2008b). Male and female mice were exposed to 0.1, 3 and 30 mg/kg GenX via oral gavage. Hepatic β‐oxidation activity was increased in males at all doses, and in females at 3 and 30 mg/kg. As in rats, enzyme activity returned to control levels after 28 days of recovery.

In addition, there is a specialized 28‐day toxicity study that focused on the evaluation of potential immunotoxicological effects of GenX (Rushing et al., 2017). In this study, male and female C57BL/6 mice were exposed to 1, 10 or 100 mg/kg GenX by oral gavage for 28 days (Rushing et al., 2017). Female mice exposed to 100 mg/kg GenX exhibited a 7% suppression of T‐cell‐dependent antibody response (TDAR) to sheep red blood cell (RBC) challenge, with no reduction in TDAR in female mice exposed to 10 or 1 mg/kg GenX (Rushing et al., 2017). No reductions were reported in male mice at 1, 10 or 100 mg/kg GenX. The study authors concluded that GenX “did not potently suppress the TDAR, even at doses that would induce high mortality.” Consistent with these conclusions, most of the other endpoints in Rushing et al. (2017) failed statistical tests for trends for dose‐response or could not be fitted to BMD models (data not shown). Importantly, the TDAR assay has been considered by some as a critical assay in determining immunotoxicity (Boverhof et al., 2014); US EPA guidance indicates that a positive TDAR response typically results in labeling an agent as immunotoxic, whereas a negative response results in a weight‐of‐evidence‐based assessment of immunotoxicity from other signs of toxicity. Overall, there is little evidence for immunotoxicity from GenX exposure.

In 2016, RIVM (Beekman, Zweers, de Vries, Janssen, & Zeilmaker, 2016) derived a tolerable daily intake value for GenX based on changes in the albumin/globulin ratio (AGR) in male rats in the 2‐year bioassay (Caverly Rae et al., 2015; Craig, 2013). The rationale provided for selecting this endpoint was concern for immunotoxicity. However, changes in the AGR alone is not a strong indicator of immunotoxicity. Overall, the change in male rats at 12 months (last time point measured) appeared minimal (0.88 in controls and 1.2 at 50 mg/kg). GenX is a PPARα activator (see above), and PPARα activators are known to affect expression of albumin and globulin with no known adverse sequelae (Caverly Rae et al., 2015). Indeed, RIVM noted that the change in AGR was consistent with other effects (e.g. liver lesions) that are “typical for peroxisome proliferators” (Beekman et al., 2016). Notably, RIVM did not cite any additional supporting evidence for immunotoxicity of GenX (Beekman et al., 2016). The dedicated immunotoxicity study by Rushing et al. (2017) does not support use of the AGR as the basis of toxicity criteria.

As in other 28‐day studies (see above), Rushing et al. (2017) demonstrated statistically significant increases in peroxisomal activity via acyl‐coenzyme A (acyl‐CoA) oxidase enzyme activity in livers of mice exposed to ≥10 mg/kg GenX. In addition, Rushing et al. measured serum levels of GenX in male and female mice after 1, 2, 3, 5, 10 and 14 days of repeated oral gavage dosing and reported no statistical difference between serum levels across time, indicating that GenX does not bioaccumulate in mice, supporting the conclusions of Gannon et al. (2016).

Subchronic 90‐day toxicity studies, conducted in accordance with OECD 408 and performed under GLP conditions, are available in both rats and mice (Haas, 2009; MacKenzie, 2010). In both species, the liver appeared to be the primary target of concern. In the mouse studies, both sexes were exposed to 0.1, 0.5 and 5 mg/kg GenX via gavage (MacKenzie, 2010). The study no‐observed‐effect level was 0.5 mg/kg/day based on liver effects in both sexes, including hepatocellular necrosis, increase in mitotic figures and increased pigment in Kupffer cells. In addition, liver/bodyweight ratios and serum liver enzymes were significantly elevated at 5 mg/kg/day. Hepatocellular hypertrophy was also increased at ≥0.5 mg/kg/day, but the study authors did not consider this lesion alone to be adverse. No other adverse effects were considered by the study authors as test article related. In female mice, serum levels of GenX did not differ statistically within dose groups after 1, 28 and 90 days of exposure, whereas serum levels were 50%‐90% higher in male mice after 28 days of exposure as compared with 1 day of exposure. The study authors concluded that steady state was reached faster in female mice than male mice (MacKenzie, 2010).

In a subchronic 90‐day oral toxicity study in rats, males were exposed to 0.1, 10 and 100 mg/kg GenX via gavage and females were exposed to 10, 100 and 1000 mg/kg by gavage (Haas, 2009). Hepatocellular hypertrophy was increased in males at ≥10 mg/kg and in females at 1000 mg/kg. Liver/bodyweight ratios were also increased in these treatment groups. There were no signs of degeneration or necrosis in these animals, and the hypertrophy was absent in the 28‐day recovery group. Serum alkaline phosphatase levels were minimally elevated in these dose groups.

Kidney/bodyweight ratios were increased in the high‐dose male (100 mg/kg) and female (1000 mg/kg) rats. Female rats in the highest dose group also exhibited evidence of diuresis and histopathological evidence of kidney injury such as renal tubular necrosis. Slight elevations in the kidney/bodyweight ratios in the 10 mg/kg male rats and 100 mg/kg female rats were not considered adverse by the study authors (Haas, 2009).

Changes in RBC parameters were observed in the high‐dose females (1000 mg/kg) and high‐dose males (100 mg/kg) (Haas, 2009). These changes included decreased RBC count, hemoglobin (Hb) and hematocrit. The RBC counts in the highest dose groups were below the historical range for the conducting laboratory. However, these effects were mitigated in the 28‐day recovery group. Red cell parameters were also slightly reduced in the 10 mg/kg male rats after 90 days of exposure; however, the study authors did not consider the minimal changes to be adverse. The study no‐observed‐adverse‐effect level (NOAEL) for male rats was 10 mg/kg/day based on regenerative anemia. The study NOAEL for female rats was 100 mg/kg/day based on regenerative anemia and decreased survival in rats exposed to 1000 mg/kg/day (Haas, 2009).

#### 3.2.3 Chronic toxicity studies

The database for GenX also includes a 2‐year combined chronic toxicity and carcinogenicity study, conducted in accordance with OECD 453 and performed under GLP conditions, in rats (Caverly Rae et al., 2015; Craig, 2013). Based on pharmacokinetic data and subchronic toxicity studies, male and female rats were administered different doses. Specifically, males received gavage doses of 0.1, 1 and 50 mg/kg and females received gavage doses of 1, 50 and 500 mg/kg (and deionized water as the vehicle control). Relative to female rats, male rats appeared more sensitive to GenX, likely due to their slower clearance (see Section 3.2.1). The liver was the primary target organ in male rats. Liver lesions included centrilobular hypertrophy, cystic focal degeneration and centrilobular necrosis (Table 1). Several non‐neoplastic lesions were observed in female rats at 500 mg/kg but not 50 mg/kg (e.g., hyperplasia in the forestomach, kidney lesions). The most sensitive effect in female rats was tubular nephropathy (see Section 3.3).

**Table 1:** Incidence of select hepatic, testicular, and pancreatic lesions in rats after chronic exposure

	Control mg/kg/day			
Males	0	0.1	1	50
Cystic focal degeneration	24/70	24/70	19/70	42/70*
Centrilobular hypertrophy	0/70	0/70	0/70	7/70*
Centrilobular necrosis	1/70	0/70	1/70	5/70*
Hepatocellular adenoma/carcinoma	2/70	2/70	1/70	3/70
Pancreas adenoma/carcinoma	0/70	1/70	0/70	5/70*
Leydig cell tumors	4/70	4/70	1/70	8/70

	Control mg/kg/day			
Females	0	1	50	500
Hepatocellular adenoma/carcinoma	0/70	0/70	0/70	13/70*

* Significantly different from control (*P* < .05). Source: Caverly Rae et al. (2015).

**Figure 2:**
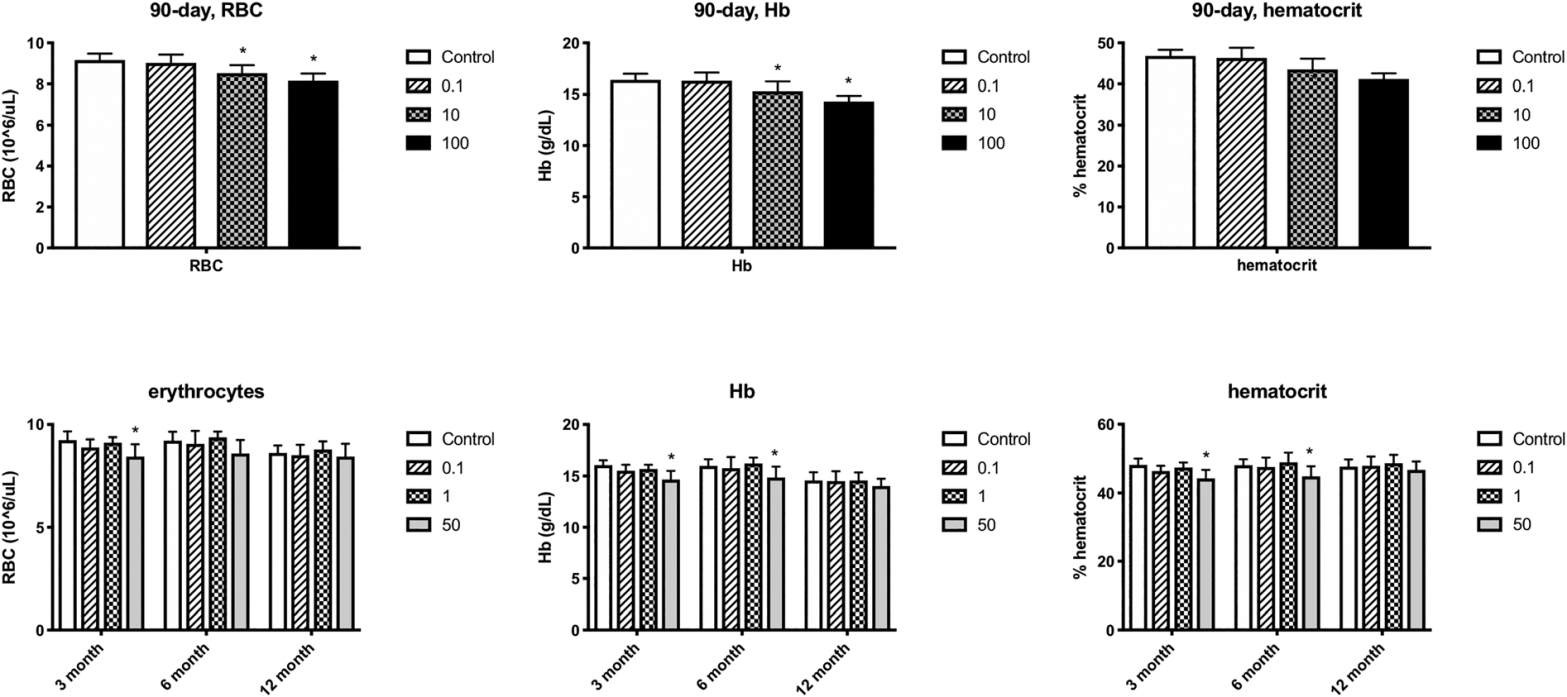
Effects of GenX on male rat red blood cell parameters. Top row: dose‐response for RBC, Hb and percentage hematocrit in 90‐day rat study. Bottom row: dose‐response for RBC, Hb and percentage hematocrit at 3, 6 and 12 months in the 2‐year rat bioassay. Data adapted from Haas (2009) and Craig (2013). Hb, hemoglobin; RBC, red blood cells

As seen in the 90‐day rat study, changes in RBC, Hb and percentage hematocrit were observed in male rats after 13 weeks of exposure. After 6 months of exposure, minor changes were only observed in Hb and hematocrit. After 12 months of exposure, no changes were apparent in any red cell parameters (Figure 2). This pattern is consistent with reported fluctuations in RBC, HB and hematocrit in young rats (Greaves, 2007). In female rats, RBC count, Hb and hematocrit were all significantly reduced at 12 months of exposure in the 500 mg/kg group.

#### 3.2.4 Genotoxicity and carcinogenicity

In vitro bacterial reverse mutation assays, in vitro mammalian chromosomal aberration tests, in vitro mammalian cell gene mutation and unscheduled DNA repair studies, were all conducted in accordance with OECD guidelines and performed under GLP conditions, and indicated that GenX was not genotoxic (Clarke, 2008; Donner, 2008; Glatt & Glover, 2008; Pant & Sly, 2007). In vivo micronucleus and chromosome aberration tests in mouse bone marrow cells were also negative (Gudi & Krsmanovic, 2007). Thus, the available data for GenX indicate that it is not genotoxic. This conclusion is consistent with other evaluations (Beekman et al., 2016; NCSAB, 2018).

In the 2‐year chronic bioassay conducted in rats, statistically significant increases in adenomas and carcinomas of the liver were observed in female rats at 500 mg/kg, but no increases were observed at 50 or 1 mg/kg (Caverly Rae et al., 2015). Peroxisomal enzyme activity was significantly elevated at 300 mg/kg in female rats, establishing a possible correlation between PPARα activation and liver tumorigenicity (see Section 3.2.6). Although GenX also increases peroxisomal enzyme activity in males, no significant elevations in liver tumors were observed in male rats treated with up to 50 mg/kg GenX (Table 1). A small but statistically significant increase in the combined incidence of pancreatic acinar cell adenomas/carcinomas were observed in male rats treated with 50 mg/kg GenX (Table 1).

Notably, many peroxisome proliferators (i.e., PPARα activators) induce a so‐called tumor triad of liver adenomas/carcinomas (mice and rats), testicular Leydig cell tumors (rats) and pancreatic acinar cell tumors (rats) (Corton, Peters, & Klaunig, 2018; Felter et al., 2018; Klaunig et al., 2003). Thus, the toxicity profile for GenX is consistent with other PPARα activators, inducing changes in the three tissues associated with the tumor triad (Table 1). There is a well‐recognized PPARα‐related, non‐genotoxic MOA for liver tumors that is generally accepted as not being relevant to humans (Corton et al., 2018; Klaunig et al., 2003). The MOA for pancreatic tumors is less understood as compared with liver tumors (Klaunig et al., 2003).

Taken together, the lack of genotoxicity, the consistent positive findings of liver hypertrophy, β‐oxidation (see Section 3.2.6), peroxisomal proliferation and lack of genotoxicity are all consistent with GenX acting through non‐genotoxic mechanisms involving PPARα activation. As such, GenX is unlikely to be a human carcinogen. This conclusion is consistent with other evaluations that focused on non‐cancer effects of GenX (Beekman et al., 2016; NCSAB, 2018).

#### 3.2.5 Reproductive and developmental toxicity

There are two OECD guideline studies for GenX that assess the potential for reproductive and developmental toxicity. In both studies, the extent of developmental toxicity was reduced bodyweight, with limited or no evidence of structural abnormalities. In both studies, reduced bodyweight in offspring was concomitantly observed with changes in the dams (e.g., liver hypertrophy). These findings suggest that the changes in fetal/pup bodyweight are secondary to maternal changes.

GenX has been studied in mice in a GLP study conducted in accordance with the OECD 421 Reproduction/Developmental Toxicity Screening Test at exposures of 0.1, 0.5 and 5 mg/kg/day via oral gavage (Edwards, 2010b). F_0_ females received daily doses of GenX 14 days before pairing and through postnatal day 20 (PND20), i.e., 53‐65 days. On PND4, eight pups (four per sex) were selected from each litter to remain in the study. On PND21, one male and one female pup from each litter were selected for the F_1_ generation, whereas the remaining pups were killed. The F_1_ generation began receiving daily doses of GenX from PND21 until they were killed on PND40. According to the study authors, no effects on reproduction were seen in mice exposed up to the highest study dose of 5 mg/kg.

**Figure 3:**
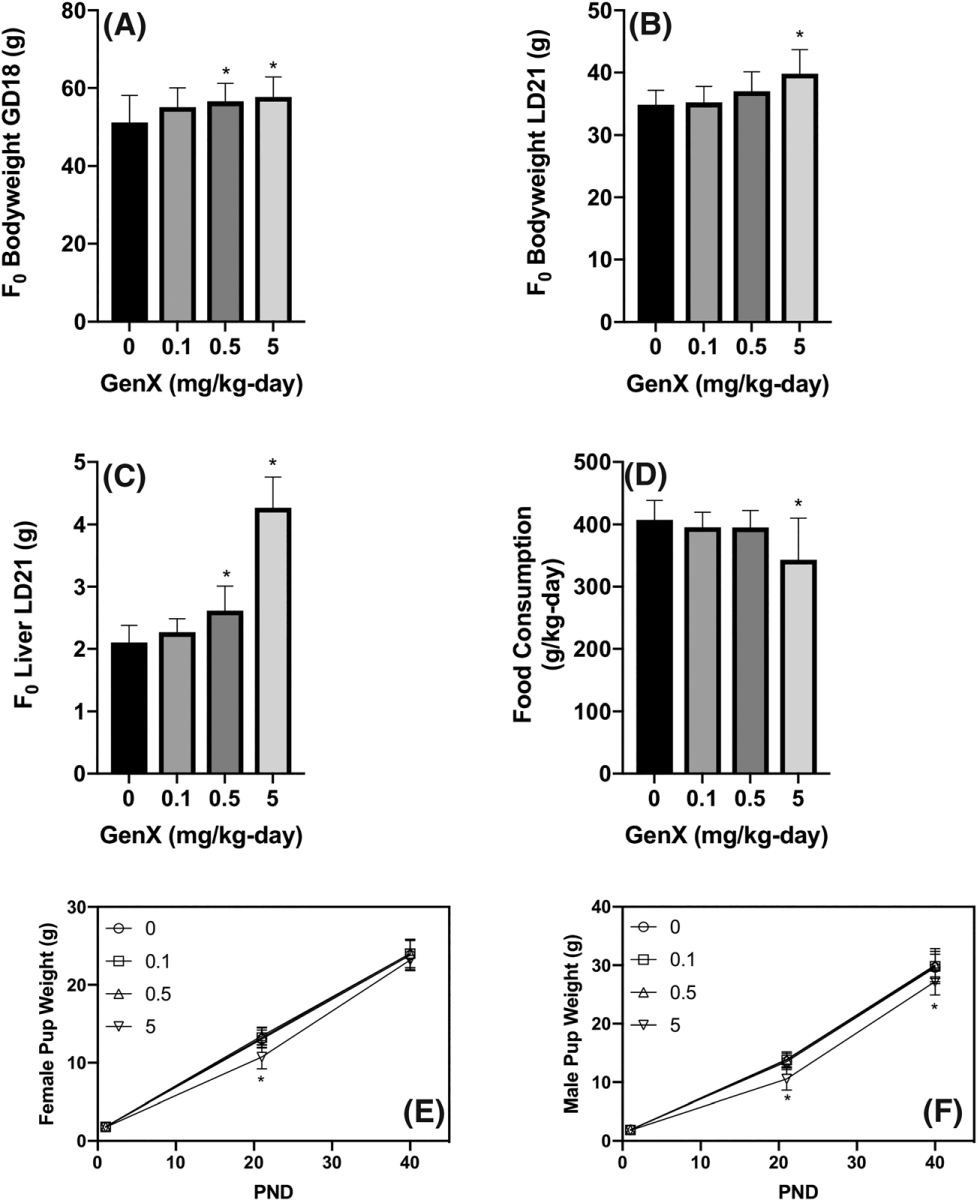
Comparison of maternal effects with developmental effects in mice. A, Maternal bodyweight on GD18. B, Maternal bodyweight LD21. C, Maternal bodyweight LD21 attributed to liver weight increases. D, Maternal food intake from LD1‐21. E, Female pup weight at PND1, PND21 and PND 40. F, Male pup weight at PND1, PND21 and PND40. Data represent mean ± SD. *Statistical significance relative to control (Dunnett's test, P < .01). Data adapted from Edwards (2010b). GD, gestational day; LD, lactational day; PND, postnatal day

At gestation day 18 and lactational day 21, the F_0_ females exhibited statistically significant increases in bodyweight that were attributed to twofold increases in liver weight (Figure 3A‐3C). In addition, hepatocellular hypertrophy was observed at ≥0.5 mg/kg and single cell necrosis at 5 mg/kg. Edwards (2010b) considered the NOAEL for maternal toxicity to be 0.5 mg/kg/day based on single cell necrosis at 5 mg/kg/day. During the first week of lactation, food consumption was significantly lower in the 5 mg/kg females on a g/animal basis, and significantly reduced over the 21‐day lactational period on a g/kg/day basis (Figure 3D). Thus, in the highest dose group, maternal liver weight was increased twofold while food consumption was lower than in other groups.

Although pup weight was similar in all groups at PND1, male and female pup weights were lower in the 5 mg/kg group at PND21, possibly due to reduced maternal food intake. From PND21 to PND40, the pups received GenX via oral gavage. By PND40, no differences in pup weight were evident in females (Figure 3E), whereas male pup weight was still slightly (<10%) reduced (albeit significantly) in the 5 mg/kg group as compared to controls (Figure 3F). It seems highly likely that the slight reductions in male pup weight were a consequence of maternal effects such as reduced food intake. Nevertheless, the study authors considered 0.5 mg/kg/day the study NOAEL for developmental toxicity.

GenX has also been studied in rats exposed to 10, 100 and 1000 mg/kg in a GLP study conducted in accordance with the OECD 414 Prenatal Developmental Toxicity Study (Edwards, 2010a). According to the study authors, the NOAEL for developmental effects was 10 mg/kg/day based on reduced fetal bodyweight at ≥100 mg/kg. As with mice, the developmental effects occurred concomitantly with changes to the livers of dams. Specifically, maternal rats in the 100 and 1000 mg/kg groups exhibited focal necrosis in the liver and increased liver weight. Food consumption was significantly reduced in the dams exposed to 1000 mg/kg GenX over the course of gestation. The study authors concluded that no external, visceral and skeletal malformations were considered test substance related or adverse. A higher litter proportion of 14th rudimentary ribs was observed in the 1000 mg/kg group but not the 100 or 10 mg/kg group. The study authors considered this effect to be treatment related, but not clearly adverse.

Taken together, these data indicate that the growth retardation observed in both species were likely related to effects concomitantly occurring in the dams. Guidance documents on developmental toxicity list several endpoints indicative of maternal toxicity: maternal mortality, bodyweight change, absolute organ and relative to bodyweight changes (particularly when supported by histopathology), and clinical chemistry such as liver enzymes (US EPA, 1991). The developmental effects induced by GenX occurred at doses that induced adverse effects in dams, suggesting that the developmental effects are possibly secondary to maternal toxicity. Nevertheless, the same US EPA guidance notes that the presence of maternal effects at doses that also affect offspring does not necessarily provide grounds for discounting the developmental effects (US EPA, 1991). As such, these effects will be discussed further in Section 3.3.

#### 3.2.6 Mode of action consideration

**Figure 4:**
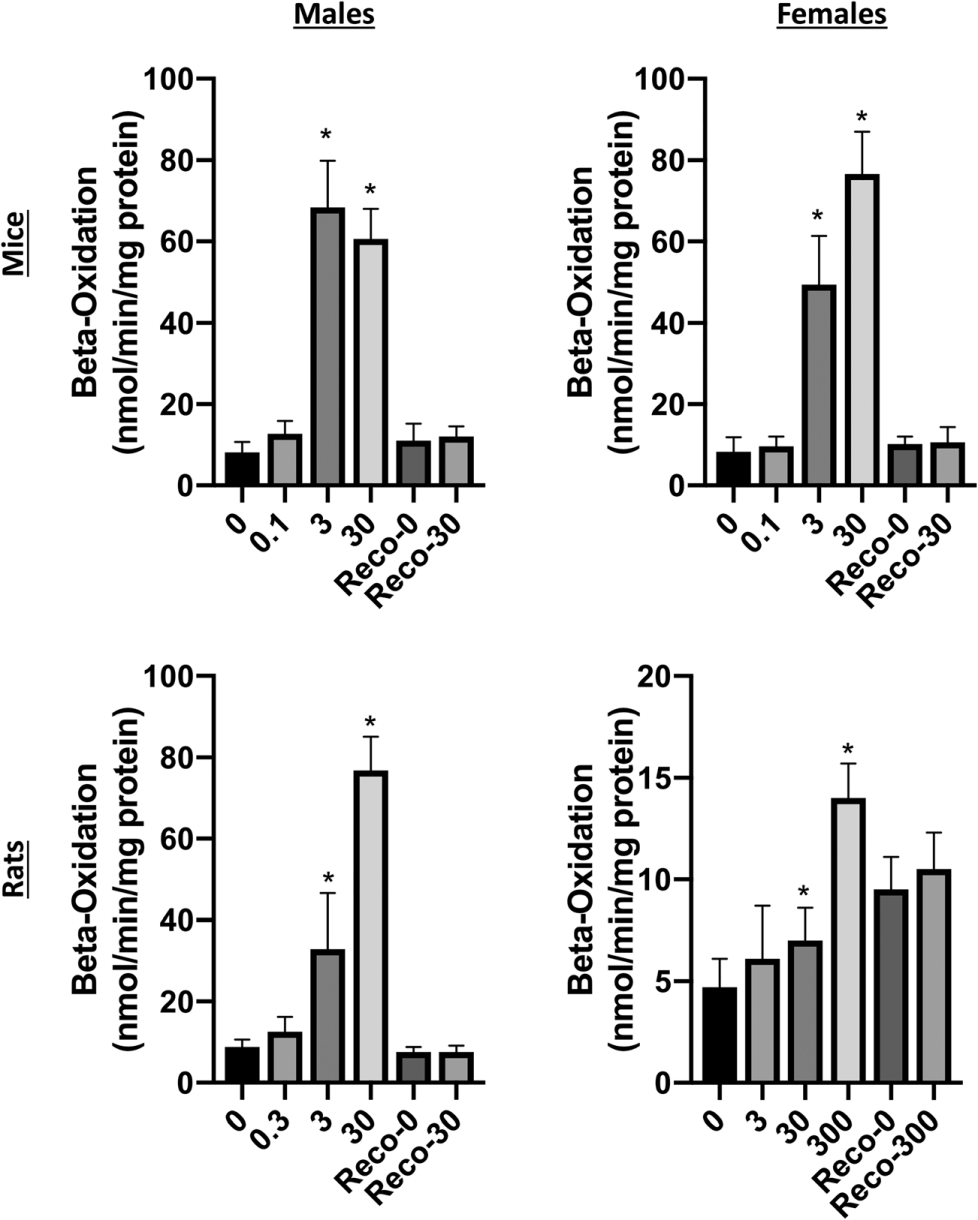
Effects of GenX on β‐oxidation enzyme activity. Data represent hepatic peroxisome β‐oxidation activity toward [^14^C]palmitoyl coenzyme A in male (left) and female (right) mice (top) and rats (bottom). *Statistical significance relative to control (Dunnett's test, P < .01). Data adapted from Haas (2008a, b)

To date, there are no formally proposed MOAs for GenX; however, the observed effects are consistent with PPARα activation. Corton et al. (2014) outline types of data that inform liver effects mediated by PPARα and other MOAs. Although Corton et al. discussed these MOA characteristics in relation to liver tumor formation, they are nonetheless useful for informing the MOA of non‐cancer liver effects. First, a PPARα‐mediated MOA does not involve direct DNA damage. As discussed in Section 3.2.4, GenX is not genotoxic. PPARα activators increase palmitoyl‐CoA oxidase or acyl‐CoA oxidase activity. Figure 4 shows the peroxisomal β‐oxidase enzyme activity in the livers of mice and rats following exposure to GenX for 28 days. The most direct evidence for PPARα activation comes from a recent study where significant pathway enrichment for PPARα signaling pathways was observed in the livers of male ICR mice exposed to 1 mg/kg GenX by oral gavage once daily for 4 weeks (Wang et al., 2017). GenX also induced other effects consistent with PPARα activation such as increased liver weight, liver hypertrophy and increased cell proliferation (evidenced by increases of mitotic figures), as well as changes in two of the three target tissues associated with the PPARα tumor triad (see Section 3.2.4).

The MOA for liver tumors induced by PFAS such as PFOA has been concluded to be mediated through PPARα activation (Klaunig, Hocevar, & Kamendulis, 2012; Li, Wang, & Klaunig, 2019). The initial key event in this MOA, i.e., PPARα activation, is plausible in humans (at some dose); however, the subsequent key events leading to cell cycle changes such as alterations in proliferation and apoptosis are specific to rodents and therefore not relevant to humans (Corton et al., 2018; Klaunig et al., 2003; Klaunig et al., 2012). Relevant for PFOA and other PFAS‐mediated PPARα activation: (1) humans express lower levels of PPARα; (2) PPARα activation is generally more sensitive in rodents than humans; (3) human PPARα activity alters lipid metabolism as opposed to the cell cycle pathways altered in rodents; and (4) PPARα activators do not increase hepatic cell proliferation in monkeys (Corton et al., 2014; Klaunig et al., 2003; Klaunig et al., 2012). These findings suggest that GenX‐induced liver tumors arising from a PPARα MOA are not likely relevant to humans.

That PPARα‐related signaling is significantly elevated in mice following exposure to 1 mg/kg GenX (Wang et al., 2017) suggests that PPARα is likely activated at lower doses. Because the Wang et al. study included only a single dose of GenX, we modeled the dose‐response for acyl‐CoA oxidase enzyme activity in male and female mice following 28 days of exposure (same data as in Figure 4) as a proxy for PPARα activation. Models fitting the female data indicated a BMD_1SD_ of 0.5 mg/kg/day (not shown). Male data could not be fit due to the steep response between 0.1 and 3 mg/kg/day and slight drop off in response at 30 mg/kg/day (see bars in Figure 4). Overall, the available data indicate that PPARα is activated in mice at <1 mg/kg/day.

Because of the sensitivity to PPARα activation in mice, we hypothesized that the single cell necrosis observed in the 90‐day mouse studies might actually be apoptosis related to increased tissue growth due to PPARα activation. In this regard, the diagnosis of single cell necrosis is often undistinguished from apoptosis. A recent expert report stated, “… apoptosis and single cell necrosis are not synonyms although previous guidance has indicated otherwise and toxicologic pathologists have often used them interchangeably … Previous guidance … recommended that the term necrosis be used to describe any morphological findings of cell death in histological sections, regardless of the pathway by which the cells died” (Elmore et al., 2016).

To better distinguish between the types of cell death, these experts characterized the difference between necrotic and apoptotic cells as follows: “The degenerating/necrotic cells … are large and swollen with pale eosinophilic cytoplasm … whereas the apoptotic cells … are small and shrunken with hypereosinophilic cytoplasm and pyknotic/fragmented nuclei” (Elmore et al., 2016).

Notably, the 90‐day GenX mouse study described the single cell necrosis as “… isolated eosinophilic bodies with occasional pyknotic nuclear fragments … thus was consistent with apoptosis” (MacKenzie, 2010). To better understand the nature of single cell necrosis observed in GenX studies, we re‐evaluated the H&E slides using the most recent diagnostic criteria (see Section 2 for details). Using these criteria, the hepatocellular death appeared to be limited to apoptosis as opposed to necrotic cell death (Table 2). A representative H&E‐stained section containing an apoptotic cell and apoptotic bodies is shown in Figure S1 (see Supporting Information). These findings are relevant for risk assessment because repeated exposure to PPARα activators increases apoptosis and sensitizes mice to pro‐apoptotic agents (Marsman, Goldsworthy, & Popp, 1992; Xiao et al., 2006). It is hypothesized that increased liver apoptosis in vivo is a feedback or mitigator of increased liver cell proliferation (Corton et al., 2014). As shown in Table 2, increases in apoptosis generally coincided with increases in mitosis.

**Table 2:** Re‐evaluation of single cell necrosis in livers of male mice exposed to 5 mg/kg GenX

Slide no.	Necrosis	Apoptosis	Mitosis
7709	0	1	0
7712	1 (3 foci necrosis*)	0	0
7715	0	2	1
7716	0	2	0
7724	0	1	0
7726	0	2	0
7730	0	2	1
7735	0	2	1
7736	0	2	1
7738	0	1	1
7739	0	1	0
7744	0	2	1
7747	0	1	1
7751	0	1	1
7759	0	2	1
7764	0	1	0
7770	0	2	0
7778	0	1	2
7780	0	2	1
7781	0	2	1
7782	0	1	0
7785	0	1	1
7801	0	1	1
7804	0	2	1
7815	0	0	0

* Such foci were observed sporadically across dose groups and was therefore not considered treatment related (see Table S3; see Supporting Information). Scoring: 0 = no evident change, 1 = minimal (present in 1‐10 hepatocytes/10, 20× fields), 2 = mild (present in 11‐40 hepatocytes/10, 20× fields), 3 = moderate (present 41‐80 hepatocytes/10, 20× fields). Data are a re‐evaluation of Edwards (2010a).

Both the carcinogenic and non‐carcinogenic effects induced by GenX are well‐known effects of PPARα activators and PFAS. There is broad consensus that tumor formation in rodents exposed chronically to peroxisome proliferators has little to no relevance for human health, as primates are less sensitive to the adverse effects of PPARα activators (Corton et al., 2018). If the tumors that result from PPARα are of no relevance to humans, then the same likely holds true for non‐neoplastic lesions. Indeed, liver hypertrophy associated with PPARα activation is not generally considered adverse as it relates to risk assessment (Hall et al., 2012). These data strongly suggest that the liver effects induced by GenX may have little to no relevance to humans. Nevertheless, we quantitatively analyzed several of the non‐neoplastic endpoints below.

### 3.3 Dose‐response analysis

Based on data in Rushing et al. (2017), GenX does not appear to present a concern for immunotoxicity, and thus data from Rushing et al. (2017) were not considered for dose‐response analysis. As discussed in Section 3.2.3, the effects on RBC parameters in males were transient. Interestingly, modeling the RBC count in the 90‐day rat study and the 13‐week time point in the 2‐year rat study resulted in BMDL_1SD_ values that differed almost 30‐fold (ranging from 0.9 to 34 mg/kg). This is consistent with admonishments not to overinterpret small changes in RBC parameters, particularly in young rats (Greaves, 2007). That no such changes in RBC count were observed after 6 and 12 months of exposure underscores the notion that these mild effects are probably not relevant for risk assessment. Reductions in red cell parameters in females at 12 months were mainly in the 500 mg/kg group. Modeling RBC counts in female rats at 12 months did not result in the lowest BMDL value for female rats (see below).

Liver lesions in subchronic mouse studies included increased liver weight, increased serum liver enzymes, hepatocellular hypertrophy, increased mitotic figures (cell proliferation) and increased apoptosis (previously diagnosed as “single cell necrosis”). These lesions are also consistent with PPARα activation as discussed above. Many of these effects are thought not to be relevant for human health risk assessment, with the possible exception of necrosis. An expert pathology workgroup concluded that hepatocellular hypertrophy was not relevant for human risk assessment in the absence of lesions such as fibrosis, steatosis and necrosis, or rather large increases in serum liver enzymes (Hall et al., 2012). It is likely that the necrosis being referred to has more regional signs of degeneration than the “single cell necrosis”. Regardless, the lesions induced by GenX appear to be apoptosis rather than necrosis. Although histopathological evidence of severe liver lesions was lacking, serum liver enzyme levels were elevated several fold. However, this increase was only apparent in the highest treatment group and may represent enzyme leakage, as opposed to frank toxicity, and therefore serum enzyme levels were not modeled.

**Figure 5:**
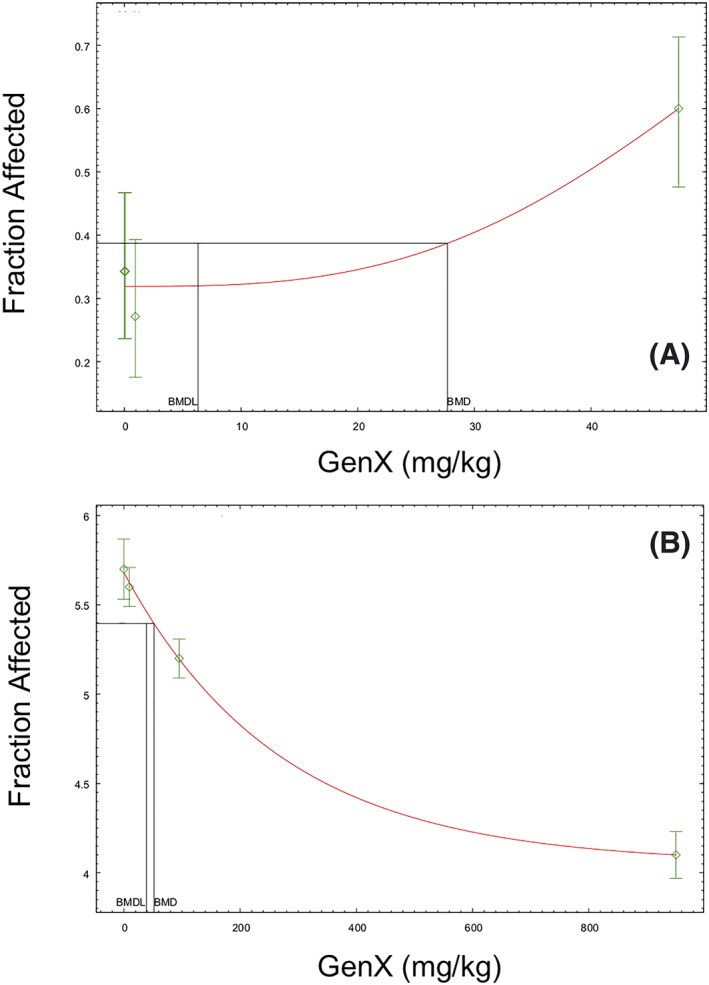
BMD modeling (frequentist models). A, Cystic focal degeneration in male rats of 2‐year bioassay (fit P = .58). B, Fetal weight in rats (exponential model; fit P = .62). Data adapted from Caverly Rae et al. (2015) and Edwards (2010a). BMD, benchmark dose; BMDL, benchmark dose and their corresponding 95% lower confidence limit values [Colour figure can be viewed at wileyonlinelibrary.com]

Because chronic bioassays are ideal for setting chronic toxicity values, the 2‐year bioassay in rats was analyzed in detail and used to benchmark all other studies and endpoints. All non‐neoplastic lesions presented in Caverly Rae et al. (2015) were modeled in male and female rats. In male rats, the liver was the most sensitive target organ. Modeling results for the three non‐neoplastic liver lesions in male rats are shown in Table 3. Although the dose‐spacing in this study is wide, the BMD models fit the data and the BMD/BMDL ratios were all less than 5, indicating a reasonable/low degree of uncertainty in the models. The lowest BMDL_10_ value was for cystic focal degeneration (6.3 mg/kg); although higher than the study NOAEL, this value is reasonable considering that there were no increases in liver lesions at 0.1 and 1 mg/kg, and only minimal increases in liver lesions at 50 mg/kg (Figure 5A). Based on the data, liver lesions would be more likely to occur closer to 50 mg/kg than 1 mg/kg. As such, 6.3 mg/kg is a reasonable (and conservative) point of departure for liver toxicity. In female rats, the BMDL_10_ values for non‐neoplastic lesions ranged from 34 to 293 mg/kg/day (data not shown); the lowest value was for kidney nephropathy.

**Table 3:** BMD modeling results

	BMD^a^	BMDL	P‐value
Male rats			
Cystic focal degeneration	28	6.3	.58
Centrilobular hypertrophy	48	29	1.0
Centrilobular necrosis	76	37	.61
Female rats			
Kidney nephropathy	44	34	.93
Developmental toxicity	52	38.5	.62

BMD, benchmark dose; BMDL, benchmark dose and their corresponding 95% lower confidence limit values; BMDS, benchmark dose software.

a BMD/L_5_ for developmental effects; BMD/L_10_ for all other endpoints. Results from BMDS 2.7.

Adverse developmental effects of GenX in rats were limited to decreases in fetal bodyweight at concentrations that also induced changes in the dams (see Section 3.2.5). Although these changes are likely secondary to maternal effects, modeling fetal bodyweight resulted in BMDL_10_ and BMDL_5_ values of 84 and 35 mg/kg/day (Figure 5B). Although the study authors did not consider the increases in 14th rudimentary cervical ribs in the highest exposure group adverse, the BMD modeling this endpoint resulted in a BMDL_1SD_ of 391 mg/kg/day (data not shown).

Similar to rats, the developmental effects of GenX in mice were limited to decreases in pup bodyweight at concentrations that also induced maternal changes (e.g. reduced food intake during lactation). PFAS such as PFOA and perfluorononanoic acid (PFNA) have been shown to cause developmental effects in wild‐type mice but not PPARα null mice (Abbott et al., 2007; Wolf, Zehr, Schmid, Lau, & Abbott, 2010). For example, PFNA caused a reduction in the number of live pups at birth, reduced survival to weaning, delayed eye opening, reduced pup weight, increased dam and pup liver weight—all at doses that did not affect maternal weight gain, implantation, litter size or pup weight at birth (Wolf et al., 2010). These effects of PFNA were not observed in PPARα null mice.

### 3.4 Deterministic reference dose derivation

Two endpoints were carried forward for RfD derivation, i.e., reduced fetal bodyweight in rats, and cystic focal degeneration in the liver of male rats exposed chronically to GenX (Table 4). For cystic focal degeneration in the liver of male rats from the 2‐year bioassay, allometric scaling was used to convert the BMDL_10_ of 6.3 mg/kg to a HED of 1.6 mg/kg. Allometric scaling accounts for both toxicokinetic and toxicodynamic differences across species and the EPA has recently recommended application of a threefold UF_A_ after allometric scaling to account for the remaining interspecies uncertainties in toxicokinetics and toxicodynamics (US EPA, 2011). While GenX is eliminated without metabolism, thus minimizing interindividual differences in disposition, a full 10‐fold UF_H_ was applied. Although the database for GenX is relatively complete, many of the studies, though publicly available, have not been published in the peer‐reviewed literature. Therefore, a threefold UF_D_ was applied. The RfD for non‐neoplastic liver lesions in male rats of the 2‐year bioassay is thus 0.02 mg/kg/day (1.6 ÷ 100) (Table 4).

**Table 4:** Candidate RfD values

Endpoint	POD (mg/kg)	HED (mg/kg)	UFs	RfD (mg/kg/day)
Fetal bodyweight (rats)	BMDL_5_ = 35	8.8	UF_A_ = 3; UF_H_ = 10; UF_D_ = 3 (total UF = 100)	0.09
Liver lesions (male rats)	BMDL_10_ = 6.3	1.6	UF_A_ = 3; UF_H_ = 10; UF_D_ = 3 (total UF = 100)	0.02
Liver lesions (male rats)	BMDL_10_ = 6.4	NA	Probabilistic approach (see text) UF_D_ = 3	0.01
	BMD_10_ = 14.2			

BMD, benchmark dose; BMDL, benchmark dose and their corresponding 95% lower confidence limit values; HED, human equivalent dose; POD, point of departure; RfD, reference dose; UFs, uncertainty factors.

For developmental toxicity in rats, allometric scaling was conducted to convert the BMDL_5_ of 35 mg/kg to HED of 8.8 mg/kg/day. The UF_A_, UF_H_ and UF_D_ are identical to those discussed above. The RfD for reduced fetal bodyweight is thus 0.09 mg/kg/day (8.8 ÷ 100) (Table 4). Because this candidate RfD is higher than the candidate RfD for liver lesions is, it did not serve as the final RfD.

### 3.5 Probabilistic reference dose derivation

There have long been calls for non‐cancer assessments to transition from traditional deterministic risk assessment approaches to risk‐based approaches more similar to those employed in cancer risk assessment (Bogdanffy et al., 2001; Clewell & Crump, 2005). Recently, there has been renewed interest in these approaches and tools made available for developing probabilistic reference values (Chiu et al., 2018; Chiu & Slob, 2015; WHO/IPCS, 2014). Aspects of this approach include using Bayesian statistics in BMD modeling, model averaging instead of selecting a single BMD model and developing probabilistic reference values. The US EPA has recently released BMDS v3.0 that includes both the frequentist BMD models used in earlier versions of BMDS and Bayesian versions of these models. Moreover, BMDS v3.0 provides model‐averaged results for the Bayesian BMD and BMDL values. With regard to probabilistic reference value development, Chiu et al. (2018) have made R code available for converting points of departure (BMDL, NOAEL, etc.) to probabilistic reference values.

**Figure 6:**
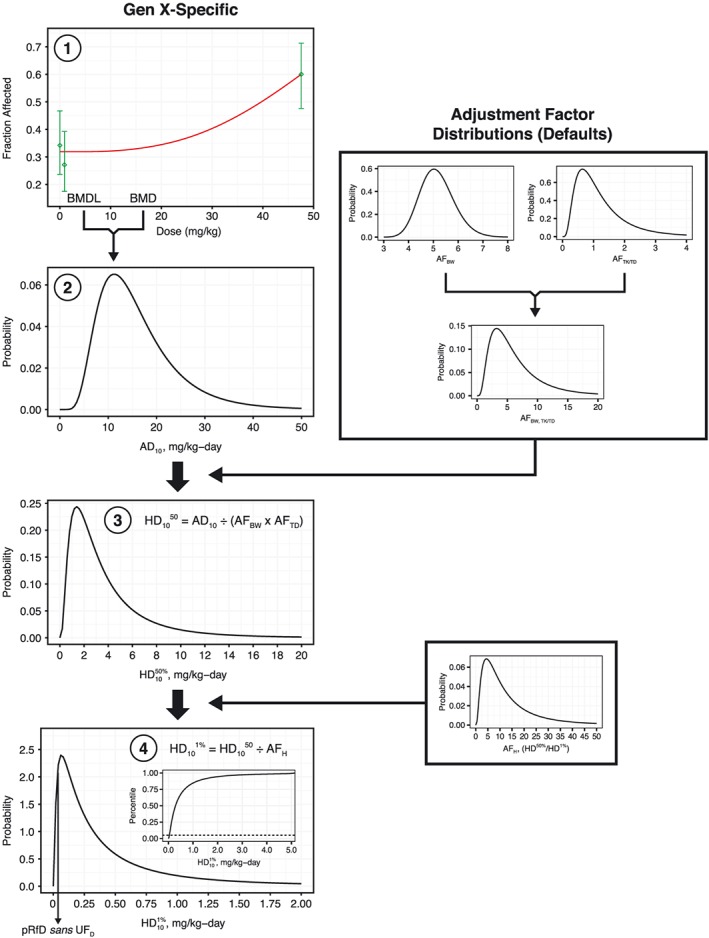
pRfD derivation. Bayesian model average BMD_10_ and BMDL_10_ values for cystic focal degeneration in male rats served as input for generating an animal distribution for 10% extra risk (AD_10_) of liver lesions (steps 1 and 2). Note: a frequentist BMD model plot from BMDS v2.7 is shown here for diagrammatic purposes only. Default adjustment factor distributions for allometric scaling factors (AF_BW_) and additional interspecies extrapolation uncertainty (AF_TK/TD_) were used to estimate equivalent human distributions of doses associated with 10% extra risk (HD_10_^50%^) of liver lesion in the typical human (step 3). Default adjustment factor distributions for human variability between the median and most‐sensitive human (defined here as the difference between the first and 50th percentiles) (AF_H_) were used to estimate a distribution for doses associated with 10% extra risk (HD_10_^50%^) of liver lesion in the most‐sensitive 1% of individuals (HD_10_^1%^) (step 4). The fifth percentile of this distribution was considered as an interim pRfD, which was then further adjusted by a threefold UF_D_. Note: the default adjustment factor distributions are those described in Chiu et al. (2018). BMD, benchmark dose; BMDL, benchmark dose and their corresponding 95% lower confidence limit values; pRfD, probabilistic reference dose [Colour figure can be viewed at wileyonlinelibrary.com]

To develop the pRfD for GenX, the incidence data for cystic focal degeneration was modeled in BMDS 3.0 to obtain BMD_10_ and BMDL_10_ values based on Bayesian model averages. The BMD_10_ and BMDL_10_ values were 14.2 and 6.4 mg/kg/day, respectively; notably, the Bayesian model average BMDL_10_ is nearly identical to the best fitting frequentist model value of 6.3 mg/kg/day derived above. As discussed in Section 2, Chiu and colleagues have proposed a more precise definition of the RfD, the so‐called HD_M_^I^, which can be interpreted for these data as the dose at which the most‐sensitive *I*% of individuals in the population have a specified *M*% extra risk of developing a non‐cancer effect. The R code to calculate the HD_M_^I^ developed by Chiu et al. (2018) was adapted to develop a pRfD from the BMDS 3.0 Bayesian model‐averaged BMD_10_ and BMDL_10_ values. Briefly, these BMD_10_ and BMDL_10_ values were used to define an estimated log‐normal distribution for the dose at which the typical rat has a 10% extra risk of developing liver lesions, the so‐called AD_10_. This dose was subsequently adjusted by probabilistic adjustment factors for allometric scaling and toxicokinetic or toxicodynamic uncertainties related to interspecies extrapolation, resulting in a distribution for the dose at which 50% of the human population has a 10% extra risk of developing liver lesions, the so‐called HD_10_^50%^ (Figure 6). The HD_10_^50%^ was further adjusted by a probabilistic human variability adjustment factor to account for the difference in equipotent doses between the median (50th percentile) and most‐sensitive 1% of the population, resulting in the HD_10_^1%^ distribution shown in the bottom of Figure 6. Chiu et al. recommended use of the fifth percentile of the HD_10_^1%^ distribution as the pRfD. However, because the Chiu et al. approach does not explicitly incorporate database uncertainty issues, Chiu and Slob (2015) recommend that adjustments related to the database “be included only after the results for the specific effect have been completed.” We therefore applied the threefold UF_D_ to the fifth percentile of the HD_10_^1%^ distribution (0.040 mg/kg/day), yielding a final pRfD of 0.01 mg/kg/day. (Note: a similar pRfD was derived using newly available tools at www.benchmarkdose.com.) This value is remarkably similar to the deterministic RfD value of 0.02 mg/kg/day.

## 4 DISCUSSION

Alternatives to long‐chain PFAS such as PFOA and PFOS were designed to avoid accumulation in mammals. Although data indicate that GenX is environmentally persistent, available toxicokinetic data for GenX indicate that it is rapidly cleared from the body and does not have the long half‐life observed for the long‐chain PFAS such as PFOA and PFOS. Human biomonitoring data collected in North Carolina shows that GenX was not found in the blood of individuals known to have GenX in tap water, indicating that GenX does not have a long half‐life like some historically used PFAS.

The analyses described herein also indicate that GenX appears to have relatively minimal toxicity, with broad effects occurring in rats only at very high doses (e.g., 500 mg/kg) and lower dose effects limited mainly to the liver and possibly mediated by PPARα activation. As already noted, humans are less sensitive to peroxisome proliferators than are rodents.

The database for GenX is robust and provides adequate data for use in developing scientifically defensible toxicity values. Further, the assessment described herein relied upon BMD modeling, which represents the preferred approach of the US EPA for dose‐response analysis. Application of both traditional deterministic and newer probabilistic BMD approaches resulted in similar RfD values. The proposed pRfD of 0.01 mg/kg/day happens to match the 0.01 mg/kg/day value originally proposed by the NCDHHS (2017). However, the NCDHHS later revised this RfD to 0.0001 mg/kg/day. The basis for the 100‐fold reduction in the RfD by the NCDHHS was driven primarily by (1) switching from a chronic bioassay to a 28‐day study, thereby necessitating a 10‐fold UF_S_, and (2) switching from rats to mice (NCSAB, 2018). It is our view that basing the RfD on PPARα‐related lesions in a subchronic study in the more sensitive rodent species (mouse) does not provide an adequate scientific basis for RfD development. Citing uncertainty about the involvement of PPARα in the liver lesions in mice, the US EPA recently released a draft assessment for GenX based on “single cell necrosis” observed in male mice exposed to GenX for ~90 days (US EPA, 2018). As shown herein, recent diagnostic criteria indicate that the single cell necrosis is better characterized as apoptosis. The apoptosis occurred concomitantly with mitosis, possibly indicating a feedback to increased cell growth and proliferation cause by PPARα activation. As previously mentioned, RIVM developed a tolerable daily intake based on the AGR in rats based on concerns for immunotoxicity (Beekman et al., 2016); however, recent immunotoxicity studies indicated weak immunotoxicity potential (Rushing et al., 2017).

The RfD developed herein should be informative for risk assessors and regulators interested in establishing acceptable levels of GenX in drinking water. As noted in the Introduction, the detection of GenX in the Cape Fear River in Eastern North Carolina (Strynar et al., 2015) and in the finished drinking water from that river (Sun et al., 2016) has raised concerns about potential drinking water exposures. The US EPA is responsible for establishing drinking water standards to control the level of contaminants in the nation's drinking water supply. This is accomplished through the promulgation of national primary drinking water regulations termed MCLs as outlined in 40 CFR, Parts 141 and 142 (US EPA, 1992). To date, the US EPA has not yet established an MCL for GenX. However, the US EPA has a very well‐developed and defined process for establishing MCLs to ensure that the nation's drinking water is safe, and that process can be applied to develop such a value for GenX. The health‐based concentration that US EPA relies on in developing MCLs is an MCLG, which is defined as the maximum level of a contaminant in drinking water at which no known or anticipated adverse effect on the health of persons would occur, allowing for an adequate margin of safety (US EPA, 2018). The MCL is set as close as feasible to the MCLG. Using the formula described in Section 2 and the RfD of 0.01 mg/kg/day, the MCLG for GenX is equal to 70 μg/L (70 ppb). This MCGL includes a default RSC of 20%. US EPA guidance indicates that RSC values can be set as high as 50% or 80% in some circumstances (US EPA, 2000). As additional exposure data become available, a departure from the default 20% RSC might be warranted. As with any criteria value, new data could result in different toxicity criteria values, either due to new points of departure or changes in the UFs as new data are generated.

## CONFLICTS OF INTEREST

Employment affiliations of the authors are shown above. ToxStrategies is a private consulting firm providing services to private and public organizations on toxicology and risk assessment issues. This work was supported by the Chemours Company FC, LLC. Chemours was given the opportunity to review the draft manuscript. The purpose of this review was for the authors to receive input on the clarity of the science presented but not on the interpretation of research results. The authors’ scientific conclusions and professional judgments were not subject to the funder's control; the contents of this manuscript reflect solely the view of the authors.

## Supporting information


**Table S1.** Histologic features of hepatocyte apoptosis
**Table S2.** Histologic Features of hepatocyte necrosis
**Table S3.** Reevaluation of Single Cell Necrosis in Livers of Male Mice Exposed to GenX
**Figure S1.** H&E stained liver section (20′ objective) from male mouse exposed to 5 mg/kg GenX exhibiting multiple apoptotic hepatocytes (arrows).

## References

[jat3812-bib-0001] Abbott, B. D. , Wolf, C. J. , Schmid, J. E. , Das, K. P. , Zehr, R. D. , Helfant, L. , … Lau, C. (2007). Perfluorooctanoic acid induced developmental toxicity in the mouse is dependent on expression of peroxisome proliferator activated receptor‐alpha. Toxicological Sciences, 98, 571–581. 10.1093/toxsci/kfm110 17488742

[jat3812-bib-0002] ATSDR . (2018). Toxicological profile for rerfluoroalkyls: Draft for public comment. *Agency for Toxic Substances and Disease Registry*.

[jat3812-bib-0003] Beekman, M. , Zweers P. , de Vries, W. , Janssen, P. , & Zeilmaker, M. (2016). Evaluation of substances used in the GenX technology by Chemours, Dordrecht. *National Institute for Public Health and the Environment*, RIVM Letter Report 2016–0174.

[jat3812-bib-0004] Bogdanffy, M. S. , Daston, G. , Faustman, E. M. , Kimmel, C. A. , Kimmel, G. L. , Seed, J. , & Vu, V. (2001). Harmonization of cancer and noncancer risk assessment: proceedings of a consensus‐building workshop. Toxicological Sciences, 61, 18–31. 10.1093/toxsci/61.1.18 11294970

[jat3812-bib-0005] Boverhof, D. R. , Ladics, G. , Luebke, B. , Botham, J. , Corsini, E. , Evans, E. , … Yang, Y. (2014). Approaches and considerations for the assessment of immunotoxicity for environmental chemicals: a workshop summary. Regulatory Toxicology and Pharmacology, 68, 96–107. 10.1016/j.yrtph.2013.11.012 24280359

[jat3812-bib-0006] Buck, R. C. , Franklin, J. , Berger, U. , Conder, J. M. , Cousins, I. T. , de Voogt, P. , … van Leeuwen, S. P. (2011). Perfluoroalkyl and polyfluoroalkyl substances in the environment: terminology, classification, and origins. Integrated Environmental Assessment and Management, 7, 513–541. 10.1002/ieam.258 21793199 PMC3214619

[jat3812-bib-0007] Caverly Rae, J. M. , Craig, L. , Slone, T. W. , Frame, S. R. , Buxton, L. W. , & Kennedy, G. L. (2015). Evaluation of chronic toxicity and carcinogenicity of ammonium 2,3,3,3‐tetrafluoro‐2‐(heptafluoropropoxy)‐propanoate in Sprague‐Dawley rats. Toxicology Reports, 2, 939–949. 10.1016/j.toxrep.2015.06.001 28962433 PMC5598527

[jat3812-bib-0008] Chiu, W. A. , Axelrad, D. A. , Dalaijamts, C. , Dockins, C. , Shao, K. , Shapiro, A. J. , & Paoli, G. (2018). Beyond the RfD: broad application of a probabilistic approach to improve chemical dose‐response assessments for noncancer effects. Environmental Health Perspectives, 126, 067009 10.1289/EHP3368 29968566 PMC6084844

[jat3812-bib-0009] Chiu, W. A. , & Slob, W. (2015). A unified probabilistic framework for dose‐response assessment of human health effects. Environmental Health Perspectives, 123, 1241–1254. 10.1289/ehp.1409385 26006063 PMC4671238

[jat3812-bib-0010] Clarke, J. J. (2008). H‐28548: In vitro mammalian cell gene mutation test (L5178Y/TK+/− mouse lymphoma assay) (DuPont). Rockville, MD: BioReliance https://hero.epa.gov/hero/index.cfm/project/page/page/1/rows/10/sort/year%20desc/format/list/project_id/2627/criteria_all/DuPont/usage_searchType/any

[jat3812-bib-0011] Clewell, H. J. , & Crump, K. S. (2005). Quantitative estimates of risk for noncancer endpoints. Risk Analysis, 25, 285–289. 10.1111/j.1539-6924.2005.00589.x 15876204

[jat3812-bib-0012] Corton, J. C. , Cunningham, M. L. , Hummer, B. T. , Lau, C. , Meek, B. , Peters, J. M. , … Klaunig, J. E. (2014). Mode of action framework analysis for receptor‐mediated toxicity: The peroxisome proliferator‐activated receptor alpha (PPARalpha) as a case study. Critical Reviews in Toxicology, 44, 1–49. 10.3109/10408444.2013.835784 24180432

[jat3812-bib-0013] Corton, J. C. , Peters, J. M. , & Klaunig, J. E. (2018). The PPARalpha‐dependent rodent liver tumor response is not relevant to humans: addressing misconceptions. Archives of Toxicology, 92, 83–119. 10.1007/s00204-017-2094-7 29197930 PMC6092738

[jat3812-bib-0014] Craig, B. S. (2013). Combined chronic toxicity/oncogenicity study 2‐year oral gavage study in rats (DuPont). Mattawan, Michigan: MPI Research, INC. https://hero.epa.gov/hero/index.cfm/project/page/page/1/rows/10/sort/year%20desc/format/list/project_id/2627/criteria_all/DuPont/usage_searchType/any

[jat3812-bib-0015] Donner, E. M. (2008). H‐27527: Bacterial reverse mutation test (DuPont). Newark, DE: DuPont https://hero.epa.gov/hero/index.cfm/project/page/page/1/rows/10/sort/year%20desc/format/list/project_id/2627/criteria_all/DuPont/usage_searchType/any

[jat3812-bib-0016] Edwards, T. L. (2010a). An oral (gavage) prenatal developmental toxicity study of H‐28548 in rats (DuPont). Ashland, Ohio: WIL Research Laboratories, LLC.

[jat3812-bib-0017] Edwards, T. L. (2010b). An oral (gavage) reproduction/developmental toxicity screening study of H‐28548 in mice (DuPont). Ashland, Ohio: WIL Research Laboratories, LLC. https://hero.epa.gov/hero/index.cfm/project/page/page/1/rows/10/sort/year%20desc/format/list/project_id/2627/criteria_all/DuPont/usage_searchType/any

[jat3812-bib-0018] Elmore, S. A. , Dixon, D. , Hailey, J. R. , Harada, T. , Herbert, R. A. , Maronpot, R. R. , … Creasy, D. M. (2016). Recommendations from the INHAND Apoptosis/Necrosis Working Group. Toxicologic Pathology, 44, 173–188. 10.1177/0192623315625859. https://hero.epa.gov/hero/index.cfm/project/page/page/1/rows/10/sort/year%20desc/format/list/project_id/2627/criteria_all/DuPont/usage_searchType/any 26879688 PMC4785073

[jat3812-bib-0019] Felter, S. P. , Foreman, J. E. , Boobis, A. , Corton, J. C. , Doi, A. M. , Flowers, L. , … Pandiri, A. (2018). Human relevance of rodent liver tumors: Key insights from a Toxicology Forum workshop on nongenotoxic modes of action. Regulatory Toxicology and Pharmacology, 92, 1–7. 10.1016/j.yrtph.2017.11.003 29113941 PMC11350555

[jat3812-bib-0020] Gannon, S. A. , Fasano, W. J. , Mawn, M. P. , Nabb, D. L. , Buck, R. C. , Buxton, L. W. , … Frame, S. R. (2016). Absorption, distribution, metabolism, excretion, and kinetics of 2,3,3,3‐tetrafluoro‐2‐(heptafluoropropoxy)propanoic acid ammonium salt following a single dose in rat, mouse, and cynomolgus monkey. Toxicology, 340, 1–9. 10.1016/j.tox.2015.12.006 26743852

[jat3812-bib-0021] Glatt, C. M. , & Glover, K. P. (2008). H‐27529: In vitro chromosome aberration test in Chinese hamster ovary cells (DuPont). Newark, DE: DuPont https://hero.epa.gov/hero/index.cfm/project/page/page/1/rows/10/sort/year%20desc/format/list/project_id/2627/criteria_all/DuPont/usage_searchType/any

[jat3812-bib-0022] Goecke‐Flora, C. M. , & Reo, N. V. (1996). Influence of carbon chain length on the hepatic effects of perfluorinated fatty acids. A 19F‐ and 31P‐NMR investigation. Chemical Research in Toxicology, 9, 689–695. 10.1021/tx950217k 8831811

[jat3812-bib-0023] Greaves, P. (2007). Histopathology of preclinical toxicity studies. London: Elsevier‐Academic Press.

[jat3812-bib-0024] Gudi, R. , & Krsmanovic, L. (2007). H‐28072: In vivo micronucleus and chromosomal aberration assay in mouse bone marrow cells (DuPont). Rockville, MD: BioReliance https://hero.epa.gov/hero/index.cfm/project/page/page/1/rows/10/sort/year%20desc/format/list/project_id/2627/criteria_all/DuPont/usage_searchType/any

[jat3812-bib-0025] Haas, M. C. (2008a). A 28‐day oral (gavage) toxicity study of H‐28397 in mice with a 28‐day recovery (DuPont). Ashland, OH: WIL Research Laboratories, LLC https://hero.epa.gov/hero/index.cfm/project/page/page/1/rows/10/sort/year%20desc/format/list/project_id/2627/criteria_all/DuPont/usage_searchType/any

[jat3812-bib-0026] Haas, M. C. (2008b). A 28‐day oral (gavage) toxicity study of H‐28397 in rats with a 28‐day recovery (DuPont). Ashland, OH: WIL Research Laboratories, LLC https://hero.epa.gov/hero/index.cfm/project/page/page/1/rows/10/sort/year%20desc/format/list/project_id/2627/criteria_all/DuPont/usage_searchType/any

[jat3812-bib-0027] Haas, M. C. (2009). A 90‐day oral (gavage) toxicity study of H‐28548 in rats with a 28‐day recovery (DuPont). Ashland, OH: WIL Research Laboratories, LLC https://hero.epa.gov/hero/index.cfm/project/page/page/1/rows/10/sort/year%20desc/format/list/project_id/2627/criteria_all/DuPont/usage_searchType/any

[jat3812-bib-0028] Hall, A. P. , Elcombe, C. R. , Foster, J. R. , Harada, T. , Kaufmann, W. , Knippel, A. , … York, M. J. (2012). Liver hypertrophy: a review of adaptive (adverse and non‐adverse) changes—conclusions from the 3rd International ESTP Expert Workshop. Toxicologic Pathology, 40, 971–994. 10.1177/0192623312448935 22723046

[jat3812-bib-0029] Hopkins, Z. R. , Sun, M. , DeWitt, J. C. , & Knappe, D. R. U. (2018). Recently detected drinking water contaminants: GenX and other per‐ and polyfluoroalkyl ether acids. Journal AWWA, 110, 13–28. 10.1002/awwa.1073

[jat3812-bib-0030] Klaunig, J. E. , Babich, M. A. , Baetcke, K. P. , Cook, J. C. , Corton, J. C. , David, R. M. , … Fenner‐Crisp, P. A. (2003). PPARalpha agonist‐induced rodent tumors: modes of action and human relevance. Critical Reviews in Toxicology, 33, 655–780. 10.1080/713608372 14727734

[jat3812-bib-0031] Klaunig, J. E. , Hocevar, B. A. , & Kamendulis, L. M. (2012). Mode of action analysis of perfluorooctanoic acid (PFOA) tumorigenicity and human relevance. Reproductive Toxicology, 33, 410–418. 10.1016/j.reprotox.2011.10.014 22120428

[jat3812-bib-0032] Kudo, N. , Suzuki, E. , Katakura, M. , Ohmori, K. , Noshiro, R. , & Kawashima, Y. (2001). Comparison of the elimination between perfluorinated fatty acids with different carbon chain length in rats. Chemico‐Biological Interactions, 134, 203–216. 10.1016/S0009-2797(01)00155-7 11311214

[jat3812-bib-0033] Lau, C. , Anitole, K. , Hodes, C. , Lai, D. , Pfahles‐Hutchens, A. , & Seed, J. (2007). Perfluoroalkyl acids: a review of monitoring and toxicological findings. Toxicological Sciences, 99, 366–394. 10.1093/toxsci/kfm128 17519394

[jat3812-bib-0034] Li, X. , Wang, Z. , & Klaunig, J. E. (2019). The effects of perfluorooctanoate on high fat diet induced non‐alcoholic fatty liver disease in mice. Toxicology, 416, 1–14. 10.1016/j.tox.2019.01.017 30711707

[jat3812-bib-0035] MacKenzie, S. A. (2010). H‐28548: Subchronic toxicity 90‐day gavage study in mice (DuPont). Newark, DE: E.I. du Pont de Nemours and company DuPont Haskell global centers for Health & Environmental Sciences https://hero.epa.gov/hero/index.cfm/project/page/page/1/rows/10/sort/year%20desc/format/list/project_id/2627/criteria_all/DuPont/usage_searchType/any

[jat3812-bib-0036] Marsman, D. S. , Goldsworthy, T. L. , & Popp, J. A. (1992). Contrasting hepatocytic peroxisome proliferation, lipofuscin accumulation and cell turnover for the hepatocarcinogens Wy‐14,643 and clofibric acid. Carcinogenesis, 13, 1011–1017. 10.1093/carcin/13.6.1011 1600604

[jat3812-bib-0037] NCDHHS . (2017). Questions and answers regarding North Carolina Department of Health and Human Services updated risk assessment for GenX (Perfluoro‐2‐propoxypropanoic acid). Raleigh, NC: North Carolina Department of Environmental Quality and North Carolina Department of Health and Human Services Secretaries’ Science Advisory Board.

[jat3812-bib-0038] NCSAB . (2018). Review of the North Carolina drinking water provisional health goal for GenX. Raleigh, NC: North Carolina Department of Environmental Quality and North Carolina Department of Health and Human Services Secretaries’ Science Advisory Board.

[jat3812-bib-0039] Pant, K. , & Sly, J. E. (2007). H‐28072: Unscheduled DNA synthesis (UDS) test with mammalian cells in vivo (DuPont). Rockville, MD: BioReliance https://hero.epa.gov/hero/index.cfm/project/page/page/1/rows/10/sort/year%20desc/format/list/project_id/2627/criteria_all/DuPont/usage_searchType/any

[jat3812-bib-0040] Rushing, B. R. , Hu, Q. , Franklin, J. N. , McMahen, R. , Dagnino, S. , Higgins, C. P. , … DeWitt, J. C. (2017). Evaluation of the immunomodulatory effects of 2,3,3,3‐tetrafluoro‐2‐(heptafluoropropoxy)‐propanoate in C57BL/6 mice. Toxicological Sciences. 10.1093/toxsci/kfw251, kfw251. 28115649 PMC6085165

[jat3812-bib-0041] Strynar, M. J. , Dagnino, S. , McMahen, R. , Liang, S. , Lindstrom, A. , Andersen, E. , … Ball, C. (2015). Identification of novel perfluoroalkyl ether carboxylic acids (PFECAs) and sulfonic acids (PFESAs) in natural waters using accurate mass time‐of‐flight mass spectrometry (TOFMS). Environmental Science & Technology, 49, 11622–11630. 10.1021/acs.est.5b01215 26392038

[jat3812-bib-0042] Sun, M. , Arevalo, E. , Strynar, M. J. , Lindstrom, A. , Richardson, M. , Kearns, B. , … Knappe, D. R. U. (2016). Legacy and emerging perfluoroalkyl substances are important drinking water contaminants in the Cape Fear River watershed of North Carolina. Environmental Science & Technology Letters, 3, 415–419. 10.1021/acs.estlett.6b00398

[jat3812-bib-0043] Thoolen, B. , Maronpot, R. R. , Harada, T. , Nyska, A. , Rousseaux, C. , Nolte, T. , … Ward, J. M. (2010). Proliferative and nonproliferative lesions of the rat and mouse hepatobiliary system. Toxicologic Pathology, 38, 5S–81S. 10.1177/0192623310386499 21191096

[jat3812-bib-0044] US EPA . (1991). Guidelines for Developmental Toxicity Risk Assessment. *Risk Assessment Forum*, EPA/600/FR‐91/001.

[jat3812-bib-0045] US EPA . (1992). National Primary Drinking Regulations; Synthetic Organic Chemicals and Inorganic Chemicals; Final Rule. *Federal Register*, 57.

[jat3812-bib-0046] US EPA . (2000). Methodology for Deriving Ambient Water Quality Criteria for the Protection of Human Health. Office of Water, US Environmental Protection Agency, EPA‐822‐B‐00‐004.

[jat3812-bib-0047] US EPA . (2011). Recommended Use of Body Weight3/4 as the Default Method in Derivation of the Oral Reference Dose. *Risk Assessment Forum*.

[jat3812-bib-0048] US EPA . (2002). A review of the reference dose and reference concentration processes. *Risk Assessment Forum*, EPA/630/P‐02/002F.

[jat3812-bib-0049] US EPA . (2012). Benchmark dose technical guidance. *Risk Assessment Forum*, EPA/100/R‐12/001. How EPA regulates drinking water contaminants. https://www.epa.gov/dwregdev/how‐epa‐regulates‐drinking‐water‐contaminants [10/16 2018]

[jat3812-bib-0050] US EPA . (2018). Human Health Toxicity Values for Hexafluoropropylene Oxide (HFPO) Dimer Acid and Its Ammonium Salt (CASRN 13252‐13‐6 and CASRN 62037‐80‐3) Also Known as “GenX Chemicals”. *U.S. Environmental Protection Agency Office of Water*, EPA‐823‐P‐18‐001.

[jat3812-bib-0051] Wang, J. , Wang, X. , Sheng, N. , Zhou, X. , Cui, R. , Zhang, H. , & Dai, J. (2017). RNA‐sequencing analysis reveals the hepatotoxic mechanism of perfluoroalkyl alternatives, HFPO2 and HFPO4, following exposure in mice. Journal of Applied Toxicology, 37, 436–444. 10.1002/jat.3376 27553808

[jat3812-bib-0052] WHO/IPCS (2014). Guidance document on evaluating and expressing uncertainty in Hazard characterization. Geneva, Switzerland: World Health Organization.

[jat3812-bib-0053] Wolf, C. J. , Zehr, R. D. , Schmid, J. E. , Lau, C. , & Abbott, B. D. (2010). Developmental effects of perfluorononanoic acid in the mouse are dependent on peroxisome proliferator‐activated receptor‐alpha. PPAR Research, 2010 10.1155/2010/282896, 1–11. PMC2948904 20936102

[jat3812-bib-0054] Xiao, S. , Anderson, S. P. , Swanson, C. , Bahnemann, R. , Voss, K. A. , Stauber, A. J. , & Corton, J. C. (2006). Activation of peroxisome proliferator‐activated receptor alpha enhances apoptosis in the mouse liver. Toxicological Sciences, 92, 368–377. 10.1093/toxsci/kfl002 16687391

