# Supplementary material for: Development of an oral reference dose for the perfluorinated compound GenX
Source: J Appl Toxicol. 2019 Jun 18;39(9):1267–82. doi: 10.1002/jat.3812 (PMC6771874; doi:10.1002/jat.3812)
Supplement: Supplementary file 1 — Table S1. Histologic features of hepatocyte apoptosis Table S2. Histologic Features of hepatocyte necrosis Table S3. Reevaluation of Single Cell Necrosis in Livers of Male Mice Exposed to GenX Figure S1. H&E stained liver section (20′ objective) from male mouse exposed to 5 mg/kg GenX exhibiting multiple apoptotic hepatocytes (arrows). [file JAT-39-1267-s001.pdf]

Supplemental Material.

Table S1. Histologic features of hepatocyte apoptosis
1. Affected hepatocytes may have condensed hypereosinophilic cytoplasm and a somewhat angular outline.<br>2. Not associated with an inflammatory response unless there is simultaneous necrosis.<br>3. May occur spontaneously with one or two affected hepatocytes present in an occasional hepatic lobule.<br>4. In standard H&E-stained sections, apoptotic hepatocytes (apoptotic bodies) are usually rounded with condensed cytoplasm.<br>5. Rounded apoptotic bodies are typically surrounded by a clear halo.<br>6. Fragments of nuclear material may be present within affected cells.<br>7. Apoptotic bodies are frequently phagocytosized by adjacent normal cells including hepatocytes and macrophages.

Table S2. Histologic Features of hepatocyte necrosis
1. Early necrotic hepatocytes are swollen.<br>2. Cytoplasm has increased eosinophilia.<br>3. Nucleus undergoing lysis, not pyknosis.<br>4. May have a minimal associated inflammatory reaction.<br>5. Can be accompanied by glycogen depletion, hydropic degeneration, fatty change, hemorrhage, and ‘‘ballooning’’ of hepatocytes.

Table S3. Reevaluation of Single Cell Necrosis in Livers of Male Mice Exposed to GenX

Slide	Group	Necrosis	Apoptosis	Mitosis
**7714**	**1**	**0**	**0**	**0**
**7717**	**1**	**0**	**0**	**0**
**7718**	**1**	**0**	**0**	**0**
**7722**	**1**	**1 (1 focus necrosis-neutrophils)**	**0**	**0**
**7723**	**1**	**0**	**0**	**0**
**7732**	**1**	**0**	**0**	**0**
**7734**	**1**	**0**	**0**	**0**
**7742**	**1**	**0**	**0**	**0**
**7750**	**1**	**1**	**0**	**0**
**7752**	**1**	**0**	**0**	**0**
**7758**	**1**	**0**	**0**	**0**
**7763**	**1**	**0**	**0**	**0**
**7765**	**1**	**0**	**0**	**0**
**7769**	**1**	**0**	**1**	**0**
**7772**	**1**	**0**	**0**	**0**
**7775**	**1**	**0**	**0**	**0**
**7788**	**1**	**0**	**0**	**0**
**7792**	**1**	**0**	**0**	**0**
**7798**	**1**	**0**	**0**	**0**
**7800**	**1**	**0**	**0**	**0**
**7803**	**1**	**0**	**0**	**0**
**7810**	**1**	**0**	**1**	**0**
**7813**	**1**	**0**	**0**	**0**
**7823**	**1**	**0**	**0**	**0**
**7825**	**1**	**0**	**0**	**0**
**7710**	**2**	**0**	**0**	**0**
**7728**	**2**	**0**	**0**	**0**
**7731**	**2**	**0**	**0**	**0**
**7737**	**2**	**0**	**0**	**0**
**7743**	**2**	**0**	**1**	**0**
**7748**	**2**	**0**	**0**	**0**
**7749**	**2**	**0**	**0**	**0**
**7754**	**2**	**0**	**0**	**0**
**7768**	**2**	**0**	**0**	**0**
**7776**	**2**	**0**	**0**	**0**
**7777**	**2**	**0**	**0**	**0**
**7779**	**2**	**0**	**0**	**0**
**7783**	**2**	**0**	**0**	**0**
**7784**	**2**	**0**	**0**	**0**
**7786**	**2**	**0**	**0**	**0**
**7787**	**2**	**0**	**0**	**0**
**7794**	**2**	**0**	**0**	**0**

7797	2	0	0	0
7805	2	0	0	0
7807	2	0	0	0
7808	2	0	0	0
7809	2	0	0	0
7811	2	0	0	0
7817	2	0	0	0
7826	2	0	0	0
7711	3	0	0	0
7720	3	0	0	0
7721	3	0	0	0
7729	*3*	0	0	0
7740	3	0	0	0
7741	3	0	0	0
7745	3	0	0	0
7746	3	0	0	0
7756	3	0	0	0
7760	3	0	0	0
7761	3	0	0	0
7762	3	0	0	0
7767	3	0	0	0
7774	3	0	0	0
7789	3	0	0	0
7790	3	0	0	0
7793	3	0	0	0
7796	3	0	0	0
7799	3	0	0	0
7802	3	0	0	0
7814	3	0	0	0
7820	3	1(Focal necrosis-neutrophils)	0	0
7822	3	0	0	0
7827	3	0	0	0
7828	3	0	0	0
7709	4	0	1	0
7712	4	1 (3 foci necrosis with neutrophils)	0	0
7715	4	0	2	1
7716	4	0	2	0
7724	4	0	1	0
7726	4	0	2	0

7730	4	0	2	1
7735	4	0	2	1
7736	4	0	2	1
7738	4	0	1	1
7739	4	0	1	0
7744	4	0	2	1
7747	4	0	1	1
7751	4	0	1	1
7759	4	0	2	1
7764	4	0	1	0
7770	4	0	2	0
7778	4	0	1	2
7780	4	0	2	1
7781	4	0	2	1
7782	4	0	1	0
7785	4	0	1	1
7801	4	0	1	1
7804	4	0	2	1
7815	4	0	0	0

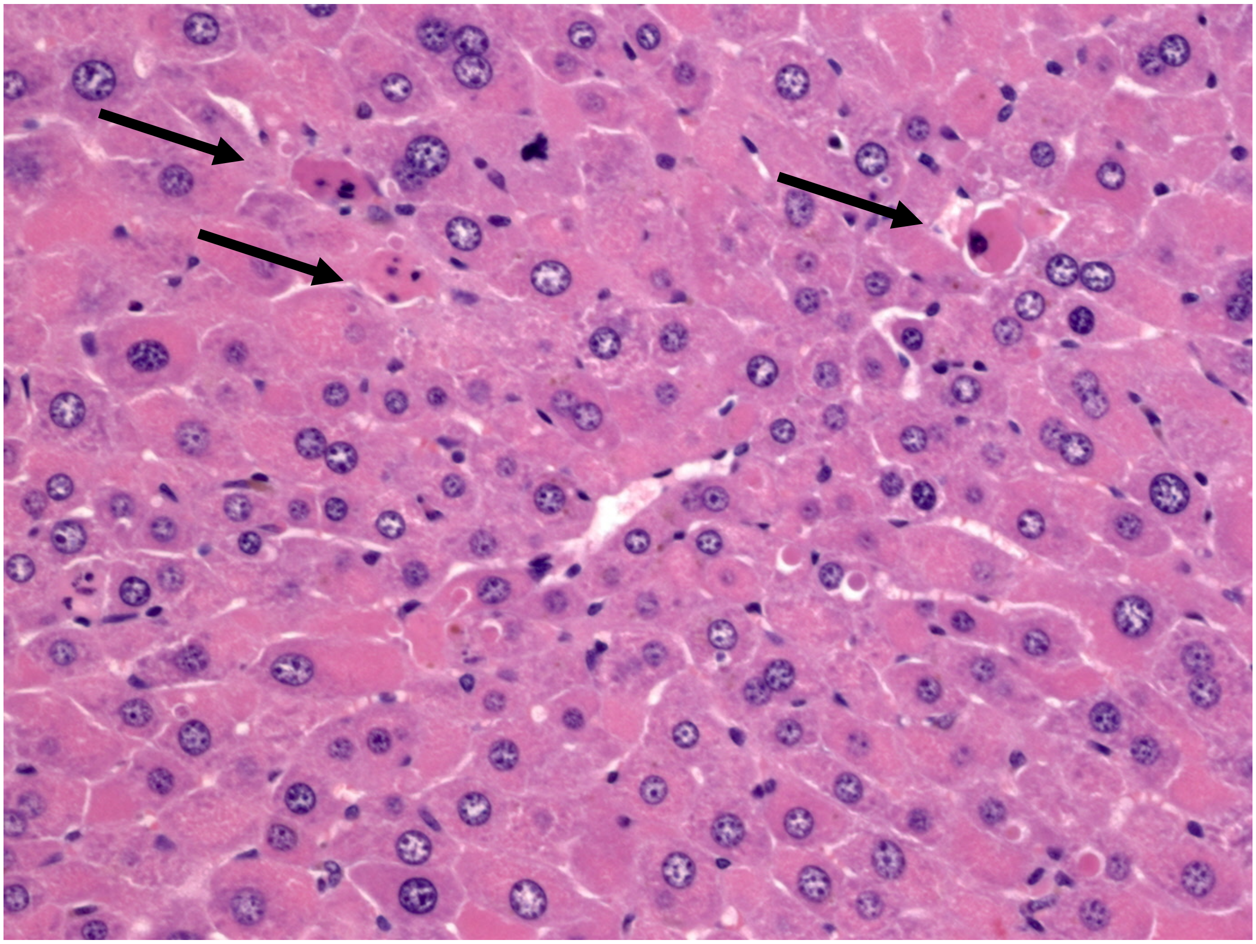

Figure S1. H&E stained liver section (20× objective) from male mouse exposed to 5 mg/kg GenX exhibiting multiple apoptotic hepatocytes (arrows).
